# A novel strategy for the manufacture of idelalisib: controlling the formation of an enantiomer†

**DOI:** 10.1039/c8ra00407b

**Published:** 2018-04-27

**Authors:** Nagaraju Mekala, Srinivasa Rao Buddepu, Sanjay K. Dehury, Krishna Murthy V. R. Moturu, Sunil Kumar V. Indukuri, Umamaheswara Rao Vasireddi, Atchuta R. Parimi

**Affiliations:** Oncology Division, Process Development Laboratories, Laurus Laboratories Limited ICICI Knowledge Park, Turkapally, Shameerpet Hyderabad-500 078 Telangana India murthy.moturu@lauruslabs.com +91 40 23480481 +91 40 30413393; Department of Organic Chemistry, Food, Drugs & Water, School of Chemistry, Andhra University Visakhapatnam-530 003 Andhra Pradesh India

## Abstract

A novel and scalable synthesis of 5-fluoro-3-phenyl-2-[(1*S*)-1-(9*H*-purin-6-ylamino)propyl]-4(3*H*)-quinazolinone, idelalisib 1, has been developed. This strategy controls the desfluoro impurity of 13 during reduction of nitro intermediate 4, and also arrests the formation of the enantiomer during cyclisation of diamide 17, without affecting the neighbouring chiral centre. This process is demonstrated on a larger scale in the laboratory and achieved good chemical and chiral purities coupled with good yields.

## Abstract 2

A novel and scalable synthesis of 5-fluoro-3-phenyl-2-[(1*S*)-1-(9*H*-purin-6-ylamino)propyl]-4(3*H*)-quinazolinone, idelalisib 1, has been developed.

## Graphical abstract

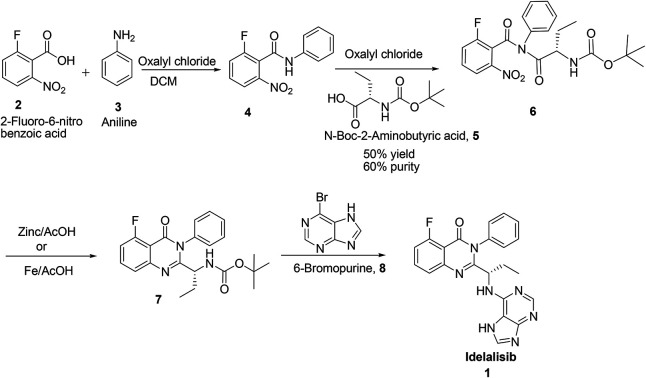

## Introduction

Quinazolinones are fused heterocyclic alkaloids and have attracted many scientists working in organic and medicinal chemistry due to their substantial and extensive range of therapeutic activities. The remarkable therapeutic activities^1^ such as antiinflammatory, anticonvulsant, antidiabetic, anticancer, antitussive, antibacterial, analgesic, hypnotic, and sedative activities coupled with exotic structural features have impelled a lot of activity in synthetic chemistry research groups towards the development of new synthetic strategies and methodologies for the synthesis of quinazolinone alkaloids. Till now approximately 200 naturally occurring quinazolinone alkaloids have been isolated from various sources.^2^

Idelalisib 1, (trade name; Zydelig), 5-fluoro-3-phenyl-2-[(1*S*)-1-(9*H*-purin-6-ylamino)propyl]-4(3*H*)-quinazolinone is a quinazolinone drug used for the treatment of chronic lymphocytic leukemia. The substance acts as a phosphoinositide 3-kinase inhibitor; More specifically, it blocks P110δ, the delta isoform of the enzyme phosphoinositide 3-kinase. The U.S. Food and Drug Administration approved idelalisib,^3^ in 2014, for the treatment of relapsed Follicular B-cell non-Hodgkin Lymphoma (FL), relapsed Chronic Lymphocytic Leukemia (CLL) and relapsed Small Lymphocytic Lymphoma (SLL). The European Medicines Agency (EMA) granted idelalisib^4^ approval for the treatment of adult patients with Chronic Lymphocytic Leukaemia (CLL) who have received at least one prior therapy and as first line treatment in the presence of 17p depletion.

As part of our research programme on the development of a series of anticancer drugs, herein we disclose a novel strategy for the synthesis of idelalisib, 1. Our approach has resulted not only in achieving almost total control of undesired enantiomer of 1, but also enhanced the yields tremendously during the cyclisation step when compared to known related reports in the literature.^5–7^

## Results and discussion

**Scheme 1:**
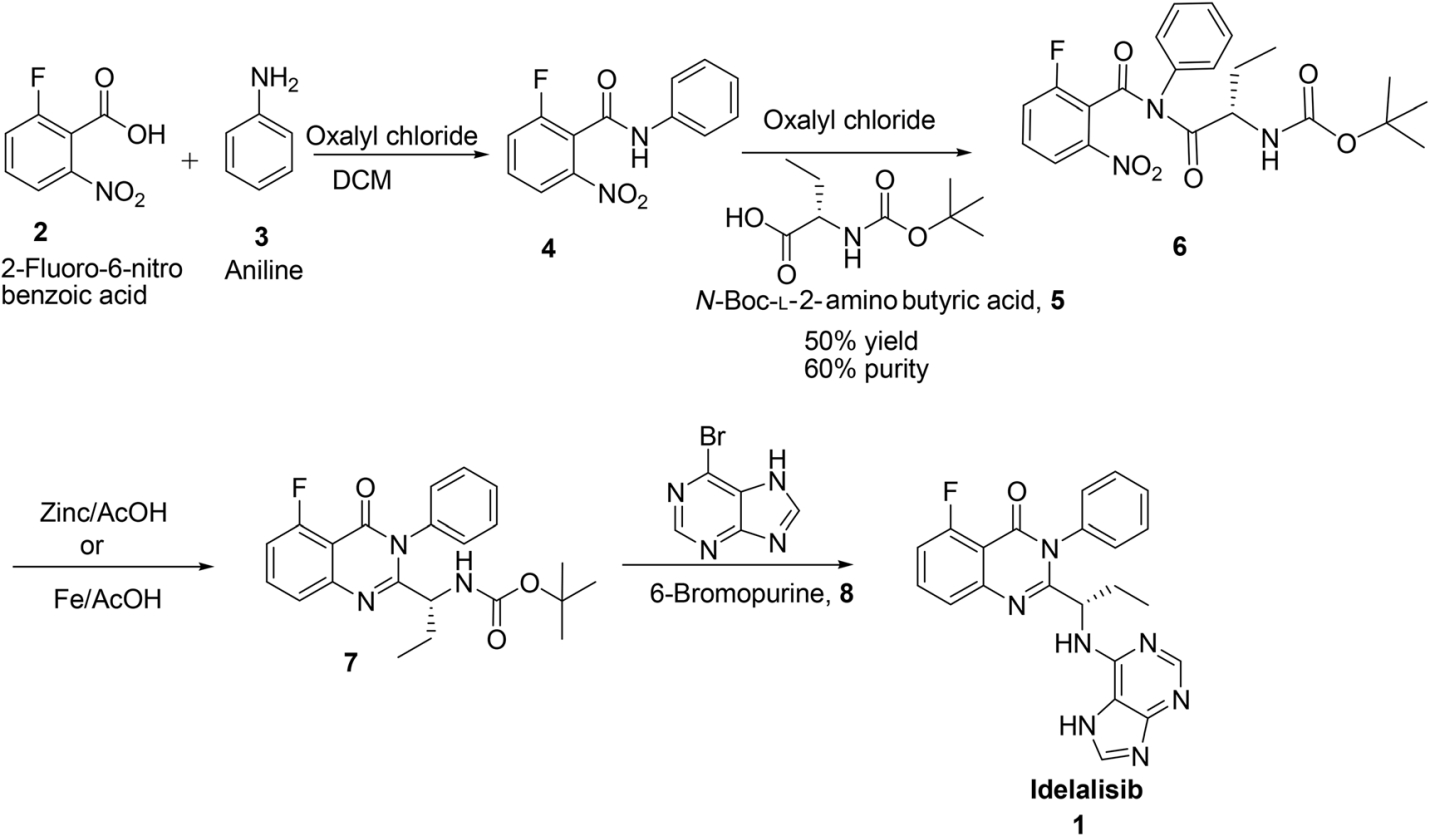
Synthesis of idelalisib, 1 reported by Icos Corporation& Shandong Kangmeile Pharmaceutical Technology Co Ltd.

Idelalisib was first developed by Icos corporation.^5^ The synthesis of idelalisib reported by Icos corporation was started with 2-fluoro-6-nitro benzoic acid (Scheme 1). Nitrobenzoic acid on treatment with oxalylchloride followed by condensation with aniline in DCM affords the corresponding 2-fluoro-6-nitro-*N*-phenylbenzamide 4. Coupling of this *N*-phenylbenzamide, 4 with *N*-Boc-l-2-amino butyric acid in presence of oxalylchloride yields *N*-boc-2-amino butyrate intermediate 6 which on reduction with Zn/AcOH affords the corresponding quinazolinone intermediate. Deprotection of Boc group followed by coupling with 6-bromopurine affords idelalisib 1. In this route reaction of *N*-Boc-l-2-amino butyric acid, 5 with nitro amide intermediate 4 is a tedious reaction. In this process only 50% conversion observed with 60% purity, due to back ward reaction. Changing the solvent medium from DCM to acetonitrile or toluene also did not give any advantage in terms of conversion and purity. In the subsequent stage, nitro group is reduced but further cyclisation is not completed to form the quinazolinone (Scheme 1).

In the second synthesis reported by Geng Fengluan *et al.*^6^ of Shandong Kangmeile Pharmaceutical Technology Co Ltd. avoided acid chloride reagent for the activation of 2-fluoro-6-nitro benzoic acid, 2 instead used CDI (Scheme 1). In the reductive cyclisation of 6, Fe/AcOH was used in place of Zn/AcOH. However, CDI is expensive to use and Fe/AcOH reagent also not viable in commercial scale.

**Scheme 2:**
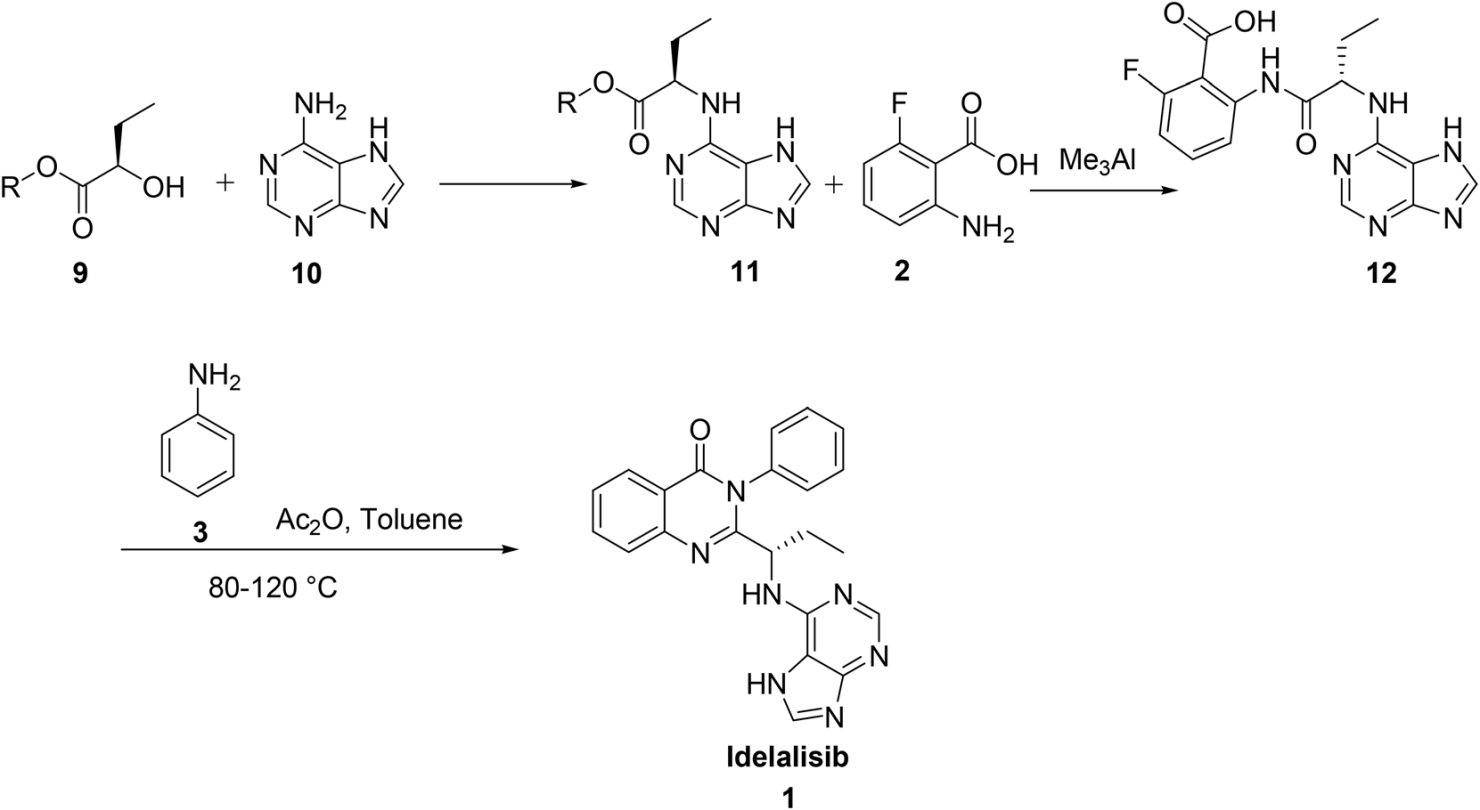
Synthesis of idelalisib, 1 reported by Suzhou Mirac Pharma Technology Co. Ltd.

The third synthesis reported by Suzhou Mirac Pharma Technology Co. Ltd^7^ is started with coupling of adenine 10 with 2-hydroxy butyrate 9 to afford the corresponding adenine derivative 11. Treatment of 11 with 2-amino-6-fluoro benzoic acid 2 in trimethylaluminium yields the corresponding acid intermediate 12. This acid intermediate on treatment with aniline, 3 in presence of acetic anhydride and toluene at high temperatures (80–120 °C) followed by cyclisation afforded idelalisib, 1. In this route coupling of aniline 3 with acid intermediate, 12 followed by cyclisation is a cumbersome reaction forming many products. The removal of aniline from the reaction mass to desired level as per ICH limit is also not achieved (Scheme 2).

**Scheme 3:**
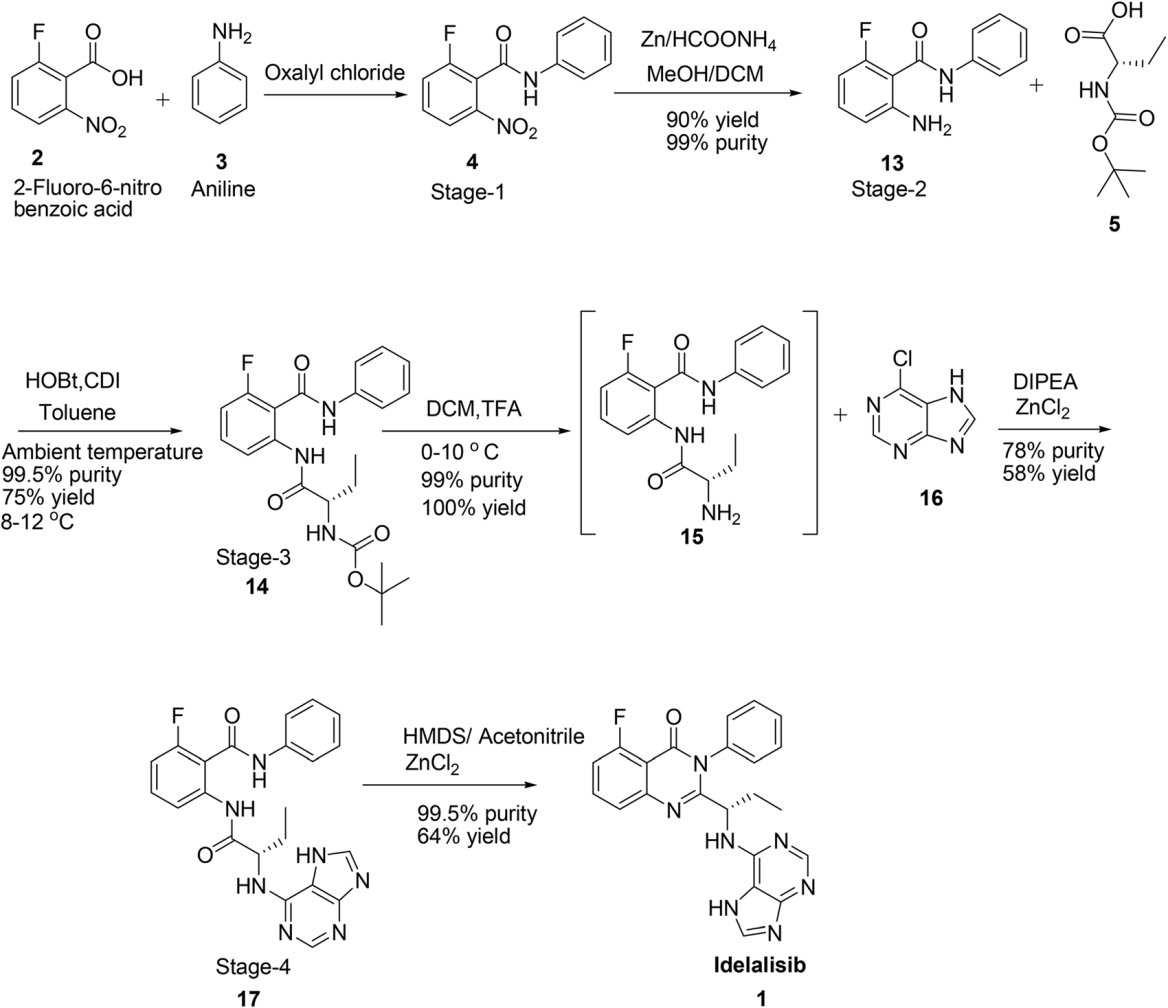
Synthesis of idelalisib, 1 by Laurus Labs Ltd.

Hence, development of idelalisib, 1 process for the manufacture requires an alternate approach to achieve ICH purity and a scalable process. As part of our process development to prepare 1 in desired cost, we have designed a novel route (Scheme 3). Unlike the innovator process (Scheme 1), *N*-Boc-l-2-amino butyric acid, 5 was coupled with 2-amino-6-fluoro-*N*-phenylbenzamide at ambient temperature and achieved >98% purities with reasonably good yield (Table 1). The reduction of nitro group for the preparation of amino intermediate is carried out at ambient temperature with conventional catalysts like 10% Pd–C (Table 1; entry 1), 5% Pd–C (Table 1; entry 2), Pt–C (Table 1; entry 3) in a 1 : 1 mixture of methanol and dichloromethane. With these catalysts desfluoro impurity is formed up to 5% level which is very difficult to separate by means of any crystallisation from the product once it is formed. Finally, this impurity is completely controlled in the reaction itself by changing the catalyst to zinc and ammonium formate^8^ to afford the 2-amino-6-fluoro-*N*-phenylbenzamide in 90% yield coupled with 99% purity (Table 1; entry 4).

**Table 1:** Reduction of 2-fluoro-6-nitro-*N*-phenylbenzamide, 4 with different catalysts

S. No.	Catalyst	Input (g)	Output (g)	Yield (%)	Purity by HPLC (% area)	
					Product	Desfluoro impurity
1^a^	10% Pd–C	110	84	87	93.5	5.6
2^a^	5% Pd–C	100	82	93	98.0	0.28
3^b^	Pt/C	10	8	91	—	0.2
4^c^	Zn/HCOONH_4_	180	142	90	99.0	Not detected

a Catalyst used: 50% wet.

b Catalyst used: 0.2 w/w to the input.

c Catalyst used: Zn: 6.0 eq. and HCOONH_4_ 7.0 eq.

In the next step for the formation of amide bond, we have attempted different coupling reagents such as benzotriazol-1-yl-oxytripyrrolidinophosphonium hexafluorophosphate (PyBOP), *N*,*N*′-dicyclohexylcarbodiimide (DCC), carbonyldiimidazole (CDI), CDI/HOBt (1-hydroxybenzotriazole) and PyBOP/HOBt for the coupling reaction of *N*-Boc-l-2-amino butyric acid with 2-amino-6-fluoro-*N*-phenylbenzamide with different solvents to achieve good yield and purity (Table 2).

**Table 2:** Coupling of 2-amino-6-fluoro-*N*-phenylbenzamide, 13 with *N*-Boc-l-2-amino butyric acid^a^

S. No.	Coupling reagent	Input (g)	Solvent	Output (g)	Yield (%)	Purity by HPLC (% area)			Remarks
						Chemical	Chiral		
						Product	Product	Enantiomer	
1	PyBOp	10	DMF	5	10	99.0	99.95	0.05	Reaction not completed (∼50% conversion observed by TLC)
2	PyBOp	10	Acetonitrile	5	32	99.0	95.0	5.0	
3	PyBOp	10	Toluene	5	28	99.0	99.9	0.1	
4	PyBOp/HOBt	10	Toluene	5	28	99.0	100	0.0	
5	DCC	10	Toluene	8	44	97.0	98.0	2.0	Reaction not completed (∼70% conversion observed by TLC)
6	CDI	10	Toluene	8	44	97.0	99.5	0.5	
7	CDI/HOBt	140	Toluene	182	75	99.5	99.99	0.01	Reaction completed by TLC

a Reagents used: 2.0 eq. of reagents used in all experiments except HOBt 1.0 eq.

As shown in Table 2, the conversion of 13 was observed ∼50% by TLC when the reaction was carried out in DMF at room temperature using PyBOP as coupling agent. However, after work up it was found that only 10% of the product was isolated with 99% purity along with control of other isomer formation (Table 2; entry 1). Changing the solvent to acetonitrile or toluene there was not much improvement in the yield but up to 5% racemisation is observed (Table 2; entry 2) in acetonitrile whereas the isomerisation was controlled to 0.10% in the latter solvent (Table 2; entry 3). Thus we decided to use toluene as solvent in the further experiments. To our good fortune racemisation is controlled completely with similar conversion and yield when 1.0 eq. of HOBt is added to PyBOP (Table 2; entry 4). Conversion is improved in both DCC (Table 2; entry 5) and CDI (Table 2; entry 6) with improved yield 44% in the same solvent but up to 2% racemisation was observed with DCC and 0.5% with CDI. Complete conversion with reasonably good yield was observed when 1.0 eq. of HOBt is added to CDI (2.0 eq.) and also totally controlled the racemisation to 0.01% (Table 2; entry 7) at lower temperature (8–12 °C). The same reaction at room temperature gives other isomer up to 0.15% level and at higher temperatures racemisation is observed up to 25%.

Deprotection of the Boc group in DCM/trifluoroacetic acid at 0–10 °C is achieved smoothly to afford the corresponding amine, 15 in quantitative yield coupled with 99% purity. This amine, 15 when coupled with 6-chloropurine, 16 in acetonitrile with diisopropylethylamine (DIPEA; 1.5 eq.) and ZnCl_2_ (1.5 eq.) at 80–90 °C for 10–12 h afforded the corresponding diamide in 58% yield with 60% purity (Table 3; entry 1). Conversion is observed only 50% by TLC when the base is changed to triethylamine but only 39% yield coupled with 98% purity with 5% of enantiomer is observed after isolation (Table 3; entry 2). Reaction accelerates when zinc chloride loading is doubled (3.0 eq.) with DIPEA base and controlled the racemisation to below 0.5% (Table 3; entry 3). Reaction does not go to completion without zinc chloride and up to 20% of other isomer is formed (Table 3; entry 4). Reaction not moved at all in other bases, such as pyridine and ammonia (Table 3; entries 5 & 6). Conversion is also observed in water but 50% racemisation is observed.

**Table 3:** Alkylation of 2-{[(2*S*)-2-aminobutanoyl]amino}-6-fluoro-*N*-phenylbenzamide, 15 with 6-chloropurine

S. No.	Base/reagent	Input (g)	Output (g)	Yield (%)	Chemical purity by HPLC (% area)	Chiral purity by HPLC (% area)		Conversion by TLC (%)
						Product	Enantiomer	
1	DIPEA/ZnCl_2_	10.0	6.0	58	60	98	2	70
2	TEA/ZnCl_2_	5.0	2.0	39	98	95	5	50
3	DIPEA/ZnCl_2_	10.0	6.0	58	78	99.5	0.5	75
4	DIPEA	10.0	3.0	29	60	80	20	30
5	Pyridine/ZnCl_2_	3.0	—	—	—	—	—	No conversion
6	NH_4_OH/ZnCl_2_	3.0	—	—	—	—	—	

Cyclisation of 17 to form the quinazoline ring closing by retaining the neighbouring chiral centre is a crucial step in the preparation of idelalisib, 1. To accomplish the cyclisation of diamide, 17 various reagents such as HMDS, HMDS/I_2_ and piperdine/I_2_ (Table 4) and solvents (Table 5) have been studied. Cyclization of diamide attempted in Zn/AcOH as per innovator process^5*a*^ at room temperature but did not result in any conversion (Table 4; entry 1). Reaction did not progress at all with different reagent/solvent systems such as PTSA/toluene, DMF, formamide and trimethyl aluminium/toluene (Table 4; entries 2–5). Reaction also attempted with ZnCl_2_/acetonitrile^9^ but found no advantage (Table 4; entry 6). When the reagent system is changed to TPP/I_2_/piperidine in acetonitrile the product was obtained in 60% purity but racemisation could not be controlled (Table 4; entry 7). When the reaction is carried out in HMDS/I_2_ in DCM only 42% yield was observed with complete racemisation (Table 4; entry 8). Complete conversion is observed but racemisation is not controlled when the DCM replaced with toluene, though 60% purity of the product is obtained (Table 4; entry 9). Reaction also attempted by replacing iodine with ZnBr_2_ but product formation was not observed at all (Table 4; entry 10).^10^ When neat reaction was carried out in HMDS/I_2_^11^ (Table 4; entry 11) 58% yield coupled with 98.9% purity was observed but racemisation could not be prevented. Finally, racemisation is completely arrested by changing the reagent to HMDS/ZnCl_2_^12^ (Table 4; entry 12) in acetonitrile and obtained the compound with good purity 99.5% in 53% yield.

**Table 4:** Cyclisation of diamide, 17 to idelalisib

S. No.	Base/reagent	Input (g)	Output (g)	Yield (%)	Chemical purity (%)	Chiral purity by HPLC (% area)	
						Product	Enantiomer
1	Zn/AcOH	0.5	—	—	—	—	—
2	pTSA/toluene	0.5	—	—	—	—	—
3	DMF	0.5	—	—	—	—	—
4	Formamide	0.5	—	—	—	—	—
5	Trimethyl aluminium/toluene	2.0	—	—	—	—	—
6	ZnCl_2_/acetonitrile	5.0	—	—	—	—	—
7	TPP/I_2_/piperidine	10.0	4.5	47	60	50	50
8	HMDS/I_2_/DCM	0.5	0.2	42	10	50	50
9	HMDS/I_2_/toluene	0.5	0.3	63	60	50	50
10	HMDS/ZnBr_2_/toluene	0.5	—	—	—	—	—
11	HMDS/I_2_	20.0	11.0	58	98.9	50	50
12	HMDS/ZnCl_2_/acetonitrile	20.0	10.0	53	99.5	99.95	0.05

**Table 5:** Selection of solvent for cyclisation of diamide, 17 to idelalisib

S. No.	Solvent	Input (g)	Output (g)	Yield (%)	Chemical purity (%)	Chiral purity by HPLC (%)	
						Product	Enantiomer
1	Toluene	40	20	48	78	50	50
2	*t*-Butanol	20	10	48	90	50	50
3	*n*-Butanol	3	2	64	80	50	50
4	2-Methyl THF	300	208	66	75	98.0	2.0
5	Acetonitrile	30	20	64	99.5	99.95	0.05

Having this result in hand further efforts were directed towards finding a suitable solvent to achieve ICH quality material coupled with good yield. Reaction in toluene afforded the product in 48% yield and 78% purity but racemised completely (Table 5; entry 1). Changing the solvent to *t*-butanol (Table 5; entry 2) or *n*-butanol (Table 5; entry 3) did not arrest the racemisation but improved yield in *n*-butanol. Reaction in 2-methyl THF afforded the similar yield as observed in *n*-butanol, however, racemisation controlled to 2% with reasonable purity (Table 5; entry 4). No conversion was observed when the reaction was carried out in water. Finally when reaction was carried out in acetonitrile racemisation was controlled to 0.05% level along with achieving high purity of 99.95% in 64% yield (Table 5; entry 5).

Finally, with all these results in hand the optimised process was executed in 150–190 g scale in the Laboratory. We observed good yield and purities in all the steps (Table 6).^13^

**Table 6:** Yields and purities of scale up experiments in all stages

Stage	Input (g)	Output (g)	Observed yield		Theoretical yield	Chemical purity by HPLC (%)
			%	w/w	w/w	
1	150	200	95	1.33	1.40	99.35
2	180	147	93	0.82	0.88	98.76
3	190	225	66	1.18	1.80	99.91
4	170	170	96	1.00	1.37	81.22
5	150	65	—	0.43	0.96	100^a^

a Chiral purity also observed in 100%.

## Experimental procedures

### Preparation of 2-fluoro-6-nitro-*N*-phenylbenzamide, 4

To a DCM solution of 2-fluoro-5-nitro benzoic acid (100 g; 540 mmol), was added catalytic amount of DMF (7.3 g, 100 mmol) and oxalylchloride (102.8 g, 810 mmol) at ambient temperature and maintained for 3 h. After completion of the reaction which was monitored by TLC, the solvent was stripped off and the obtained residue was dissolved in 1,4-dioxane (80 mL) and kept a side. In another RBF having 250 mL 1,4-dioxane was added aniline (50.3 g; 541 mmol) and aq. sodium bicarbonate (90 g in 250 water) solution. To this aniline solution was added the above 1,4-dioxane solution at 0–5 °C, maintained at ambient temperature for 90 min. After completion of the reaction which was monitored by TLC, the reaction mass was quenched into water (1500 mL) and stirred for 2 h at ambient temperature. The obtained pale yellow colour solids were filtered, washed with water to afford 2-fluoro-6-nitro-*N*-phenylbenzamide in 86% yield and 99.0% purity which was proceed to next step without further purification. ^1^H NMR data were identical with those reported in the literature.^5*a*,*b*^

### Preparation of 2-amino-6-fluoro-*N*-phenylbenzamide, 13

To a mixture of methanol (500 mL) and dichloromethane (500 mL), was added 2-fluoro-6-nitro-*N*-phenylbenzamide (100 g; 385 mmol), zinc powder (140 g; 2154 mmol) and then slowly added aq. ammonium formate solution (dissolved 170 g ammonium formate in 300 mL water) and stirred the reaction at ambient temperature for 4–5 h. The progress of the reaction was monitored by TLC. After completion of the reaction zinc salts were filtered, washed with dichloromethane (500 mL). The solvent from filtrate was stripped off completely under vacuum and added water (1000 mL) to the obtained residue. The emitted white-pale pink colour solids were filtered washed with water to afford 2-amino-6-fluoro-*N*-phenylbenzamide in 90% yield. The product used as such to next step without further purification. Mp by DSC: 112.58 °C; ^1^HNMR (300 MHz, DMSO-*d*_6_) *δ* 10.27 (s, 1H), 7.63 (m, 2H), 7.33 (t, *J* = 7.5 Hz, 2H), 7.14 (m, 2H), 6.75 (d, *J* = 8.1 Hz, 1H), 6.42 (m, 2H), 5.7 (s, 2H); ^13^CNMR (75 MHz, DMSO-*d*_6_) *δ* 167.87, 163.03, 161.80, 158.58, 149.36, 139.15, 131.41, 128.50, 123.50, 120.25, 111.38, 108.60, 101.90; MS: *m*/*z* 231(M^+^ + H).

### Preparation of 2-{[(2*S*)-2-amino(butoxycarbonyl)butanoyl]amino}-6-fluoro-*N*-phenylbenzamide, 14

To a toluene (500 mL) solution of *N*-Boc-l-2-amino butyric acid (100 g; 493 mmol), was added 2-fluoro-6-amino-*N*-phenylbenzamide (100 g; 435 mmol) cooled to 8–15 °C and added carbonyldiimidazole (140.8 g, 869 mmol) followed by 1-hydroxybenzotriazole (117.4 g, 868.6 mmol) and stirred for 30–32 h. After completion of the reaction which was monitored by TLC, the mass was quenched into water (500 mL). The obtained precipitate was filtered, washed with water. The wet material was leached with acetonitrile (300 mL), filtered and dried to afford off-white coloured 2-{[(2*S*)-2-amino(butoxycarbonyl)butanoyl]amino}-6-fluoro-*N*-phenylbenzamide, 14 in 67% yield. Mp by DSC: 167.48 °C; ^1^HNMR (300 MHz, DMSO-*d*_6_) *δ* 10.54 (s, 1H), 9.93 (s, 1H), 7.99 (d, 1H, *J* = 8.4 Hz), 7.74 (m, 2H), 7.52 (m, 1H), 7.32 (m, 2H), 7.12 (m, 2H), 3.89 (broad s, 1H), 1.74 (m, 1H), 1.55 (m, 1H), 1.30 (s, 9H), 0.86 (t, *J* = 7.2 Hz, 3H); ^13^CNMR (75 MHz, DMSO-*d*_6_) *δ* 171.50, 161.41, 160.69, 157.43, 155.70, 138.60, 137.70, 131.75, 128.50, 124.07, 120.93, 119.96, 117.56, 115.75, 111.12, 78.33, 56.99, 28.00, 24.38, 10.54; MS: *m*/*z* 414(M^+^ − H).

### Preparation of 2-{[(2*S*)-2-amino(7*H*-purin)butanoyl]amino}-6-fluoro-*N*-phenylbenzamide, 17

To a solution of 2-{[(2*S*)-2-amino(butoxycarbonyl)butanoyl]amino}-6-fluoro-*N*-phenylbenzamide (100 g; 241 mmol) in dichloromethane (200 mL), was added trifluoroacetic acid (200 mL) and stirred for 3 h at ambient temperature. After completion of the reaction which was monitored by TLC, added water (2000 mL) to the reaction mass and adjusted the pH to neutral with saturated sodium bicarbonate solution. The obtained precipitate was filtered, washed with water and dried to afford 2-{[(2*S*)-2-aminobutanoyl]amino}-6-fluoro-*N*-phenylbenzamide, 15. Mp by DSC: 195.46 °C; ^1^HNMR (300 MHz, DMSO-*d*_6_) *δ* 10.64 (s, 1H), 8.13 (brs, 1H), 7.14 (d, *J* = 8.4 Hz, 2H), 7.56 (m, 2H), 7.50 (m, 2H), 7.32 (m, 1H), 7.13 (m, 1H), 3.96 (t, *J* = 6 Hz, 1H), 1.74 (q, 2H), 0.89 (t, *J* = 7.5 Hz, 3H); ^13^CNMR (75 MHz, DMSO-*d*_6_) *δ* 168.43, 160.62, 158.42, 158.01, 157.28, 139.01, 135.70, 131.10, 128.70, 123.81, 120.59, 119.40, 115.29, 112.75, 53.66, 24.54, 8.82; MS: *m*/*z* 316(M^+^ + H). The obtained compound was dissolved in acetonitrile (500 mL) and added 6-chloro purine (39 g; 253 mmol), zinc chloride (98.4 g, 722 mmol) followed by diisopropylamine (36.5 g, 361 mmol) and maintained at 80–100 °C for 10–12 h. After completion of the reaction which was monitored by TLC, the reaction mass was quenched into water (2000 mL), stirred for 3 h and filtered. The obtained crude product was purified by leaching with methanol (200 mL) and THF (200 mL), filtered and dried to afford 2-{[(2*S*)-2-amino(7*H*-purin)butanoyl]amino}-6-fluoro-*N*-phenylbenzamide, 17 in 60% yield. Mp by DSC: 267.99 °C; ^1^HNMR (300 MHz, DMSO-*d*_6_) *δ* 12.96 (s, 1H), 10.47 (s, 1H), 10.10 (s, 1H), 8.14 (t, *J* = 10.2 Hz, 2H), 7.89 (d, *J* = 8.1 Hz, 2H), 7.82 (d, *J* = 5.4 Hz, 1H), 7.48 (m, 2H), 7.24 (m, 2H), 4.79 (br s, 1H), 1.96 (m, 2H), 0.94 (t, *J* = 7.2 Hz, 3H); ^13^CNMR (75 MHz, DMSO-*d*_6_) *δ* 171.41, 161.05, 160.46, 157.20, 154.02, 151.96, 150.00, 139.18, 138.35, 137.34, 131.50, 131.37, 128.57, 123.93, 119.71, 118.30, 116.50, 111.37, 111.06, 56.34, 24.39, 10.70; MS: *m*/*z* 434(M^+^ + H).

### Preparation of 5-fluoro-3-phenyl-2-[(1*S*)-1-(9*H*-purin-6-ylamino)propyl]-4(3*H*)-quinazolinone, 1(idelalisib)

To a solution of 2-{[(2*S*)-2-amino(7*H*-purin)butanoyl]amino}-6-fluoro-*N*-phenylbenzamide, 50 g, 115 mmol), in acetonitrile (250 mL) was added zinc chloride (51 g, 374 mmol), hexamethyldisilazane (55.9 g, 347 mmol) and maintained the reaction mass at reflux for 4 h. After completion of the reaction which was monitored by TLC, the reaction mass was quenched into water (1250 mL), stirred for 2–3 h, filtered and washed with water. The obtained crude product was dissolved in DCM (500 mL), adjusted pH to 2.0–3.0 with 10% aq. HCl. The DCM phase separated and fresh DCM (500 mL) was added to the aq. phase and neutralised with aq. sodium carbonate. The obtained product is filtered, washed with water, dried and finally recrystallized from acetonitrile (500 mL) to afford a white coloured 5-fluoro-3-phenyl-2-[(1*S*)-1-(9*H*-purin-6-ylamino)propyl]-4(3*H*)-quinazolinone, 1 in 60% yield and 99.9% purity. The spectral data of the compound were identical with those reported in the literature.^5*a*,*b*^

## Conclusion

In summary, we successfully developed a facile, novel and scalable strategy to prepare idelalisib. Cyclisation of the diamide without affecting the neighboring chiral center which is a challenging step was achieved smoothly. We further optimized this process and demonstrated the preparation in larger scale in the laboratory.

## Conflicts of interest

There are no conflicts to declare.

## Supplementary Material

RA-008-C8RA00407B-s001

## References

[cit1] Hart D. J. (2010). ARKIVOC.

[cit2] Kshirsagar U. A. (2015). Org. Biomol. Chem..

[cit3] https://www.accessdata.fda.gov/drugsatfda_docs/label/2014/206545lBl.pdf

[cit4] http://www.ema.europa.eu/ema/index.jsp?curl=/pages/medicines/human/medicines/00384 3/human_med_001803.jsp

[cit5] (a) Fowler K. W. , Huang D., Kesicki E. A., Ooi H. C., Oliver A., Ruan F., Treiberg J. and Deep Puri K., *US Pat.*, 8980901, 2015

[cit6] Guangyong L. , Xiaojun L., Mingwei F. and Fengluan G., CN patent, 104130261, 2014

[cit7] Xu X. , CN patent, 104262344, 2015

[cit8] Channe Gowda D., Mahesh B., Gowda S. (2001). Indian J. Chem..

[cit9] Yadav M. R., Shirude S. T., Parmar A., Balaraman R., Giridhar R. (2006). Chem. Heterocycl. Compd..

[cit10] Fujita S. , Reddy P. Y. and Toru T., *US Pat.*, 5965746, 1999

[cit11] Lanting X., Jiang Y., Dawei M. (2012). Org. Lett..

[cit12] Kshirsagar U. A., Mhaske Santosh B., Argade Narshinha P. (2007). Tetrahedron Lett..

[cit13] Dehury S. K. , Mekala N., Srinivasa Rao B., Sunil Kumar I. V. and Umamaheswara Rao V., PCT International Application, 191608A1, 2017

