# Supplementary material for: A novel strategy for the manufacture of idelalisib: controlling the formation of an enantiomer
Source: RSC Adv. 2018 Apr 27;8(28):15863–9. doi: 10.1039/c8ra00407b (PMC9080089; doi:10.1039/c8ra00407b)
Supplement: RA-008-C8RA00407B-s001 [file RA-008-C8RA00407B-s001.pdf]

Electronic Supplementary Material (ESI) for RSC Advances.
This journal is © The Royal Society of Chemistry 2018

^{1}HNMR Spectrum of Stage-1

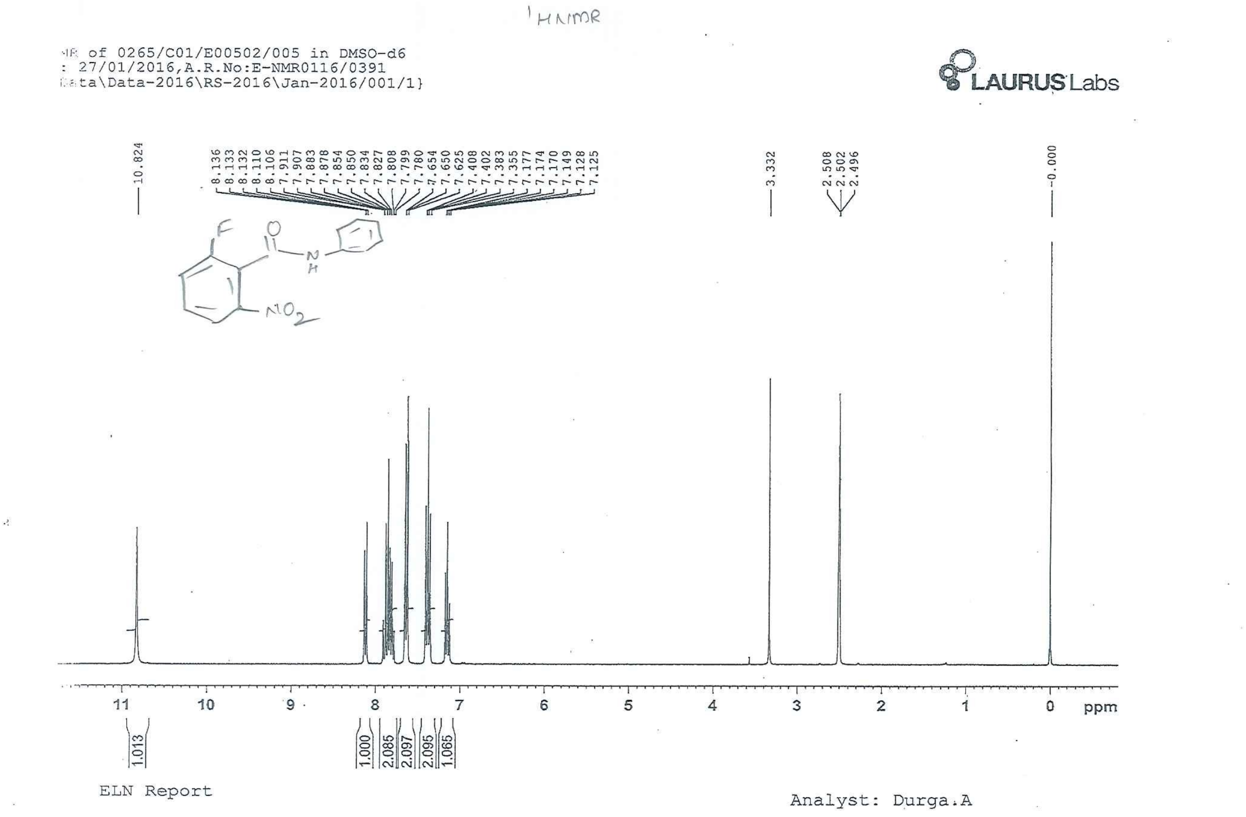

13CNMR Spectrum of Stage-1

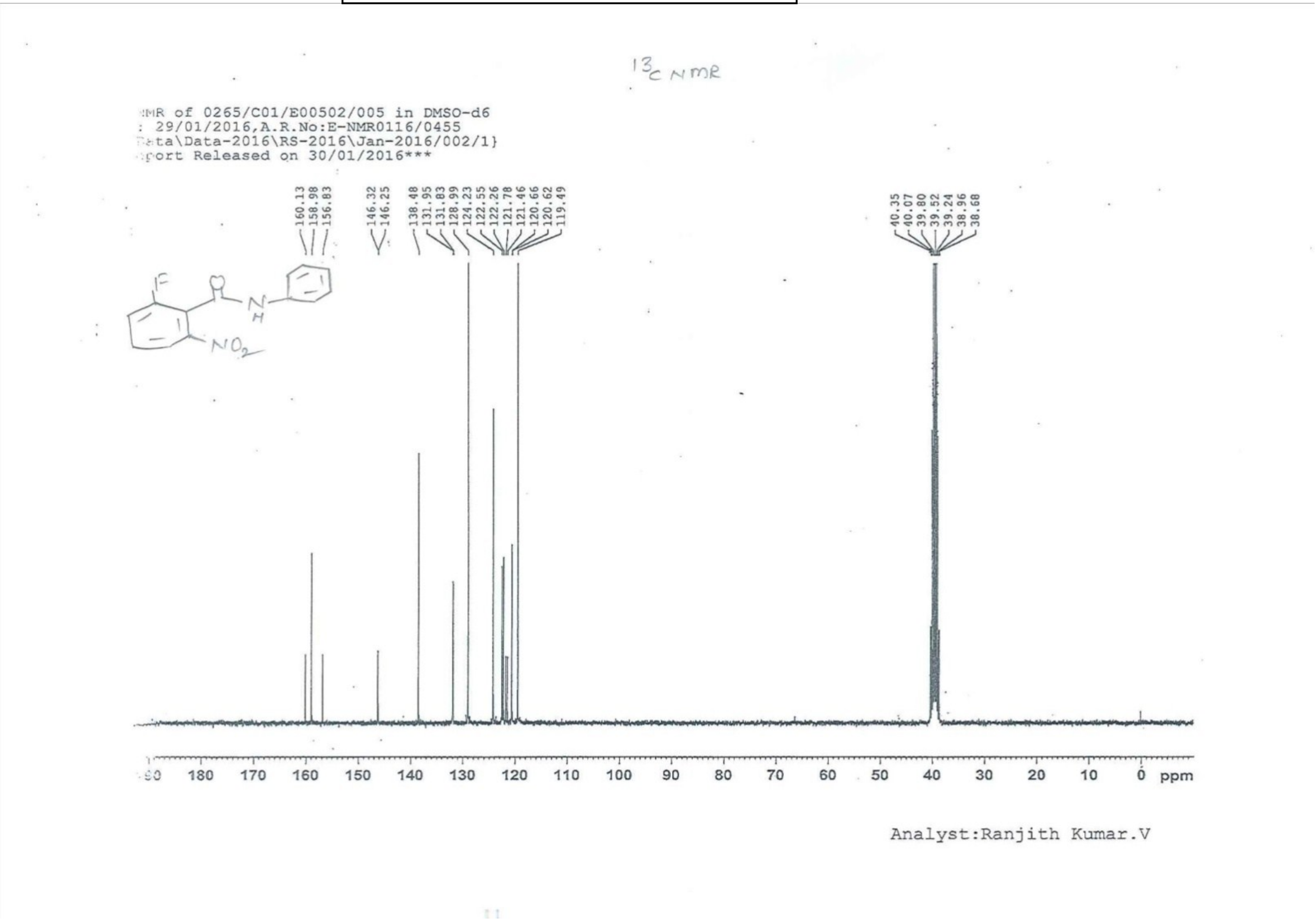

Mass spectrum of Stage-1

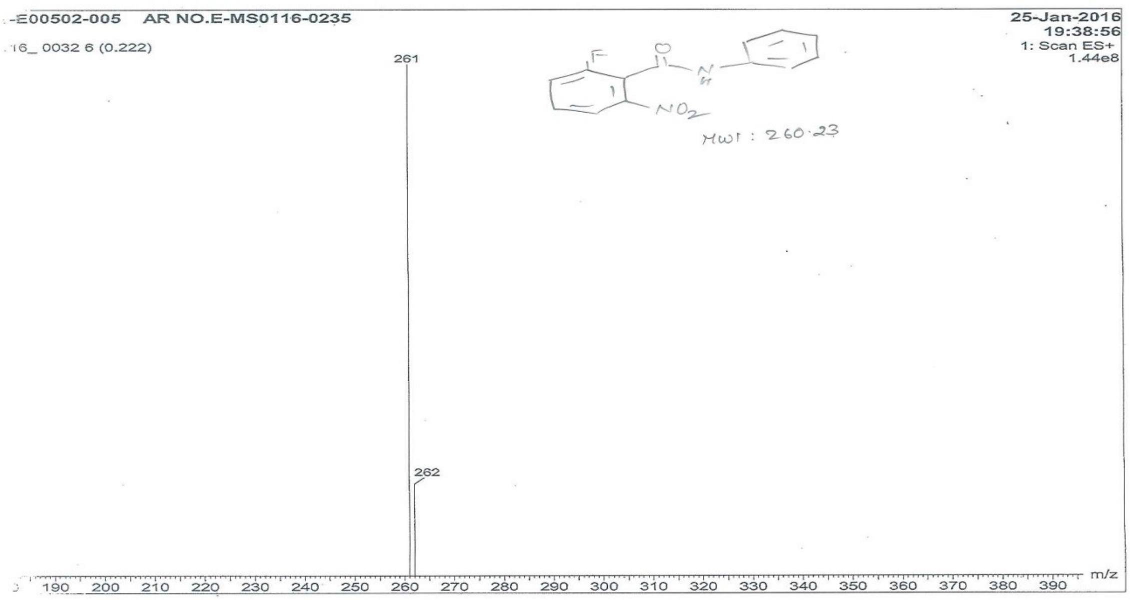

DSC Thermogram of Stage-1

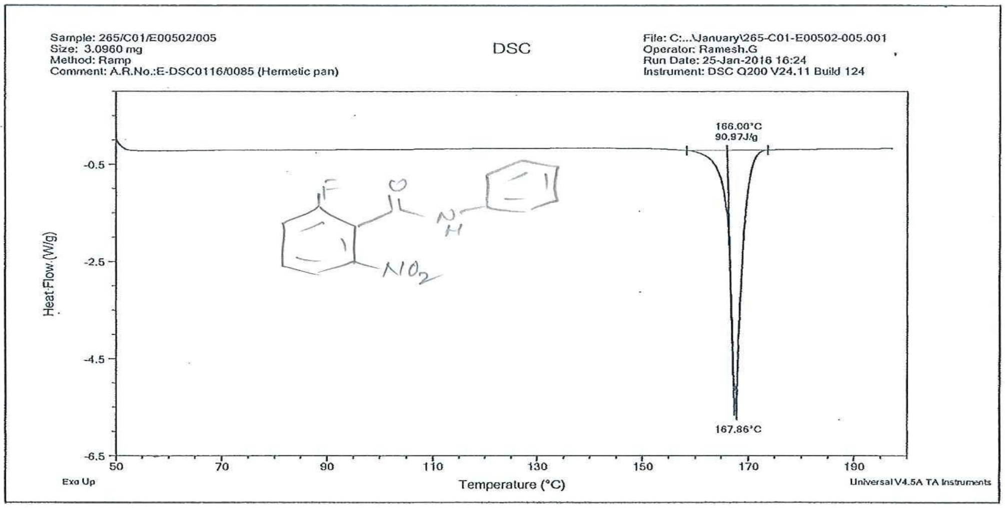

Method Log:

1: Equilibrate at 50.00°C

2: Ramp 10.00°C/min to 200.00°C

3: End of method

Peak Integration

Start	Onset	Maximum	Stop	Area
°C	°C	°C	°C	J/g
158.27	166.00	167.86	173.76	90.97

ELN

HPLC Chromatogram of Stage-1

LAURUS Labs

Analytical Development

SAMPLE INFORMATION

Sample Name: 0265/C01/E00502/005
Sample Type: Unknow n
Vial: 26
Injection #: 1
Injection Volume: 10.00 ul
Sample Set Id 1709
Sample Set Name: 300116_INT_073
Project Name: HYD1 AD-Generics\0265 Jan 2016
Result Id 1765 Result Set Id

Instrument Method Id 1663
Acquired By: divyakumarteam
Date Acquired: 1/30/2016 7:39:06 PM IST
Date Processed: 2/3/2016 8:47:17 AM IST
Processing Method: 0265_Pro
Processing Method Id 1761
Proc. Chnl. Descr.: W2489 ChA 245nm
System Name LL_AD_LC_073
Softw are: Empow er 3 Softw are Build 3471

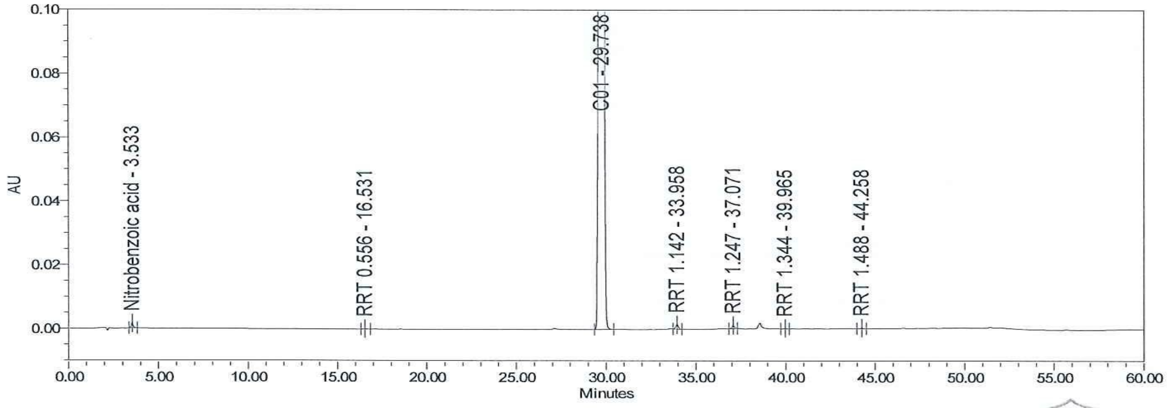

Peak Results

	Name	RT	Area (µV*sec)	% Area
1	Nitrobenzoic acid	3.53	13417	0.0517
2	RRT 0.556	16.53	1155	0.0045
3	C01	29.74	25898749	99.8368
4	RRT 1.142	33.96	14432	0.0556
5	RRT 1.247	37.07	10115	0.0390
6	RRT 1.344	39.96	1316	0.0051
7	RRT 1.488	44.26	1913	0.0074

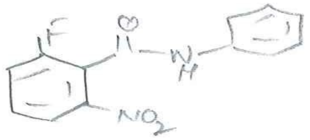

^{1}HNMR Spectrum of Stage-2

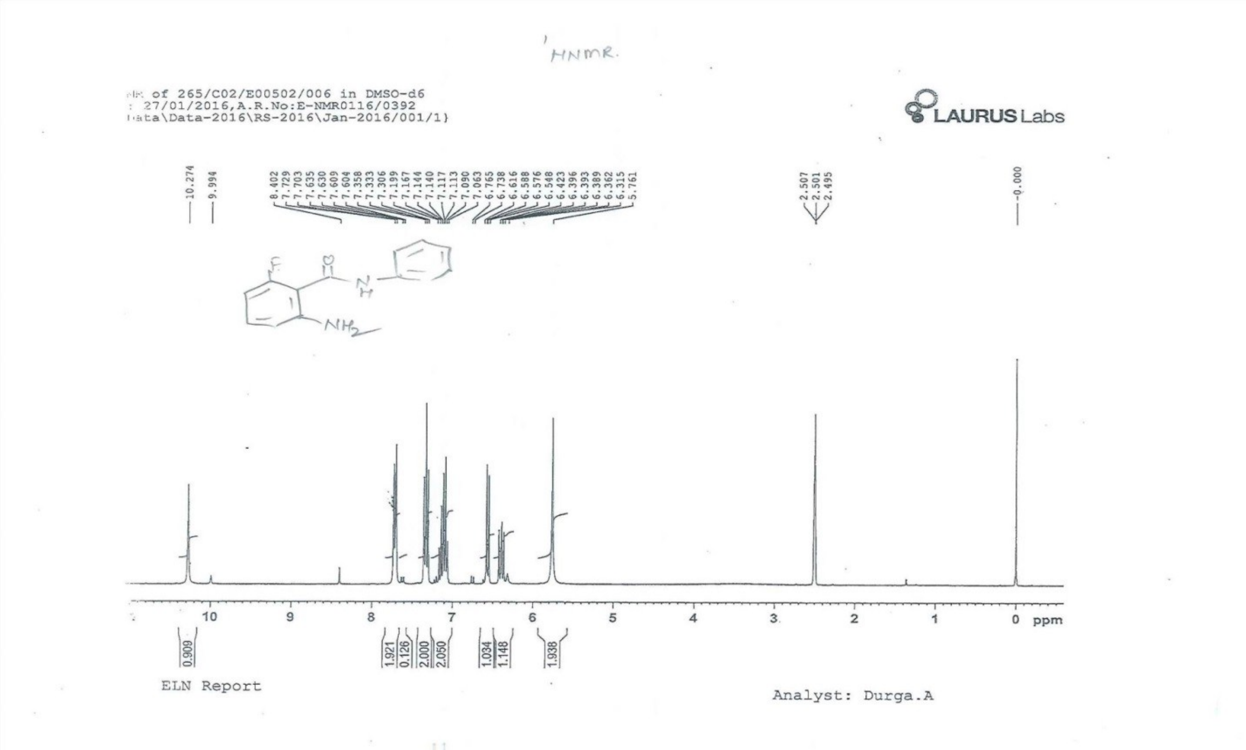

^{13}CNMR Spectrum of Stage-2

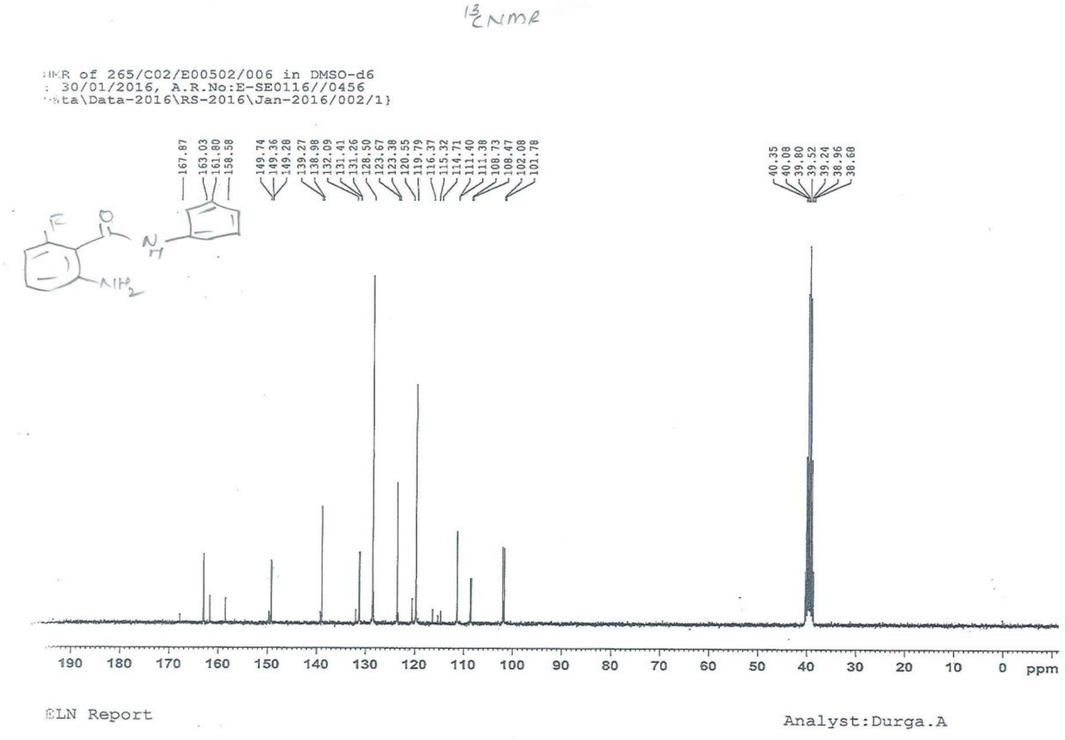

Mass Spectrum of Stage-2

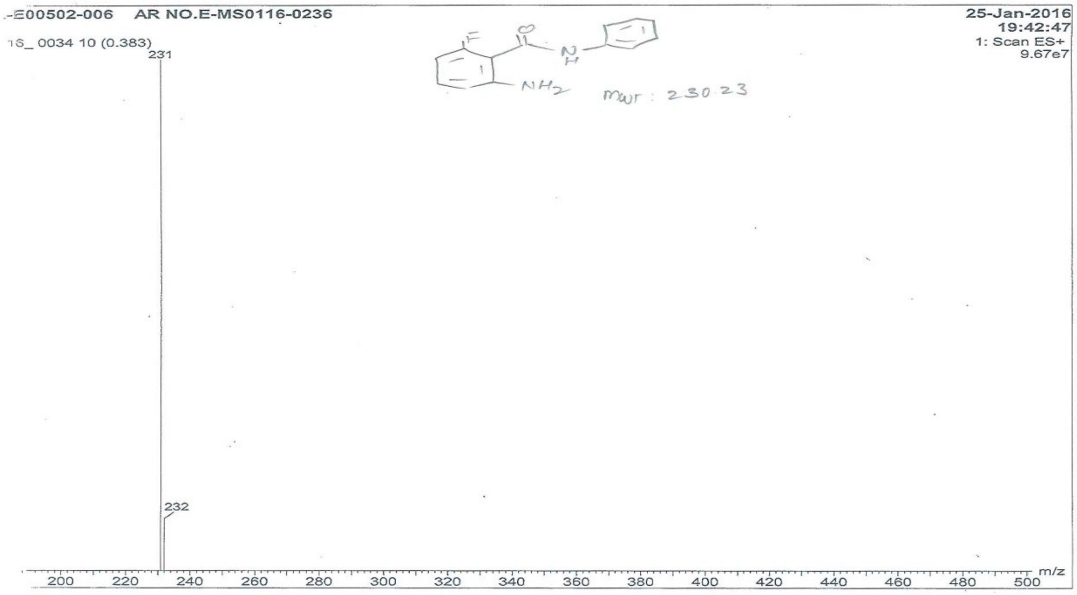

DSC Thermogram of Stage-2

LAURUS Labs

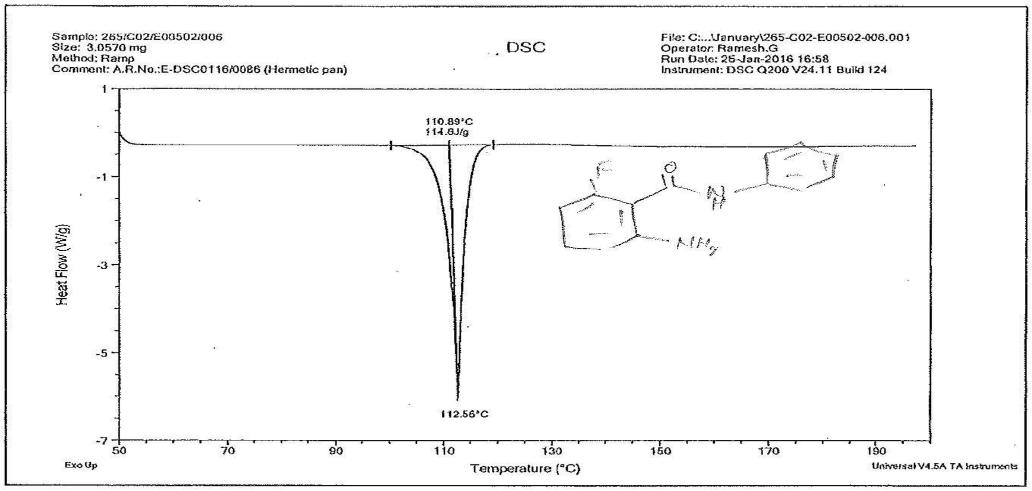

Method Log:

1: Equilibrate at 50.00°C

2: Ramp 10.00°C/min to 200.00°C

3: End of method

Peak Integration

Start	Onset	Maximum	Stop	Area
°C	°C	°C	°C	J/g
100.16	110.89	112.56	119.20	114.6

ELN

HPLC Chromatogram of Stage-2

LAURUS Labs **Analytical Development**

SAMPLE INFORMATION

Sample Name:	0265/C02/E00541/164	Method Id ****	
Sample Type:	Unknown	Acquired By:	Anjaneyuluteam
Vial:	20	Date Acquired:	07/16/2016 19:16:55 IST
Injection #:	1	Date Processed:	07/16/2016 20:52:57 IST
Injection Volume:	10.00 ul	Processing Method	0265_PRoc
Run Time:	60.0 Minutes	Processing Method Id 3744	
Sample Set Name	0265_C01_60Min_160716	Proc. Chnl. Descr.:	Detector A 247nm
Sample Set ID:	3627	System Name AL_AD_LC_39_142	
Project Name	HYD1_AD-Generics\0265_JULY_	Empower 3 Software Build 3471 SPs Installed:	
Result Id 3761	Result Set Id	Feature Release 2 DB ID: 2487711617	

Auto-Scaled Chromatogram

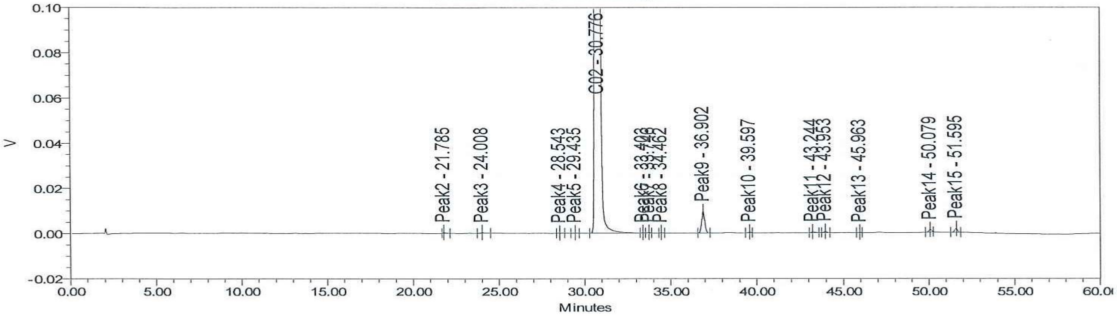

Peak Results

Name	RT	Area	% Area	RT Ratio
Peak2	21.79	4115	0.02	0.71
Peak3	24.01	6523	0.04	0.78
Peak4	28.54	979	0.01	0.93
Peak5	29.43	2587	0.02	0.96
C02	30.78	16992282	98.91	1.00
Peak6	33.40	1450	0.01	1.09
Peak7	33.75	2658	0.02	1.10
Peak8	34.46	1241	0.01	1.12
Peak9	36.90	120476	0.70	1.20
Peak10	39.60	3509	0.02	1.29
Peak11	43.24	6144	0.04	1.41
Peak12	43.95	5797	0.03	1.43
Peak13	45.96	1479	0.01	1.49
Peak14	50.08	11373	0.07	1.63
Peak15	51.59	19354	0.11	1.68

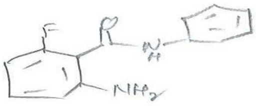

Sample Name: 0265/C02/E00541/164
Date Acquired: 07/16/2016 19:16:55 IST

Signature / Date

Page: 1 of 1

^1HNMR Spectrum of Stage-3

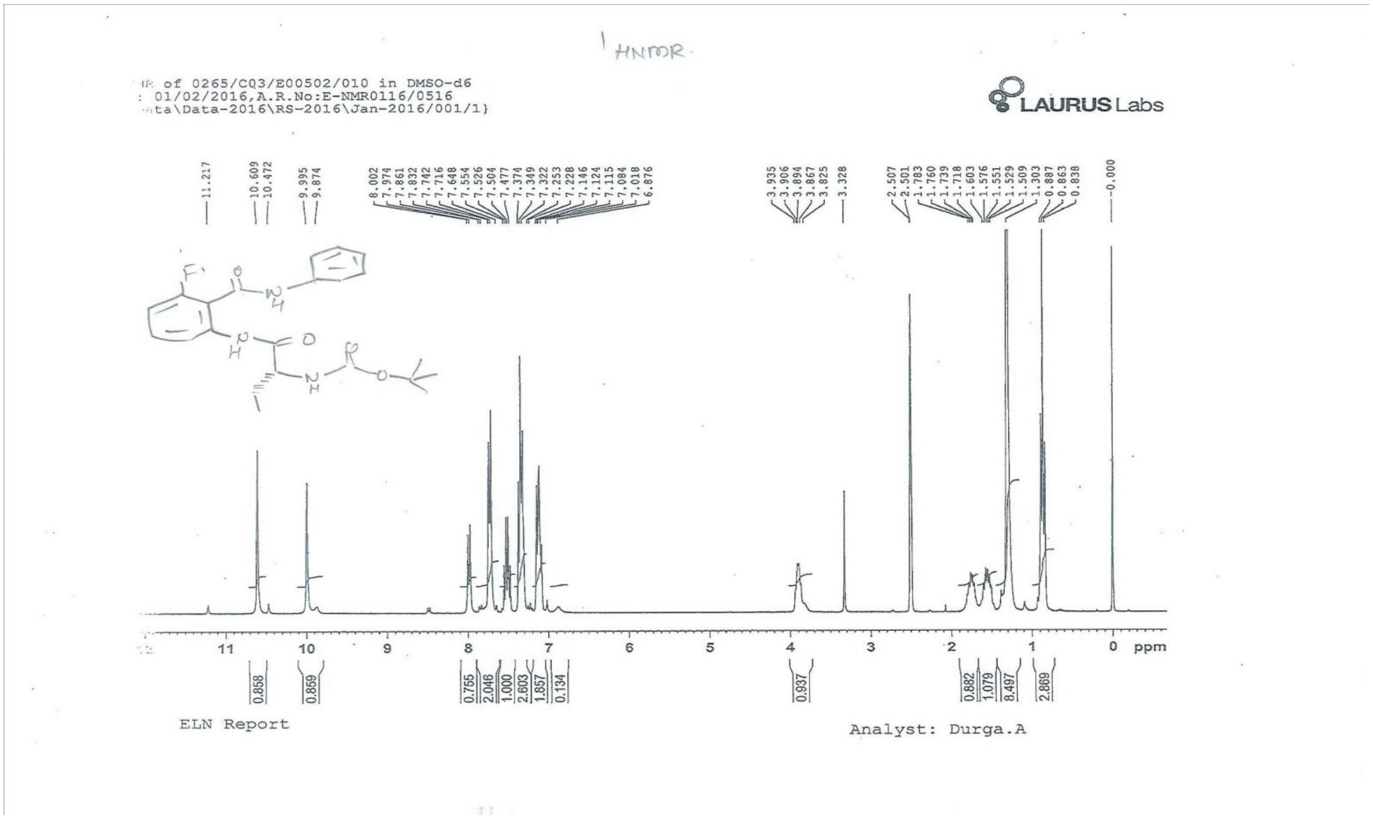

[13] CNMR Spectrum of Stage-3

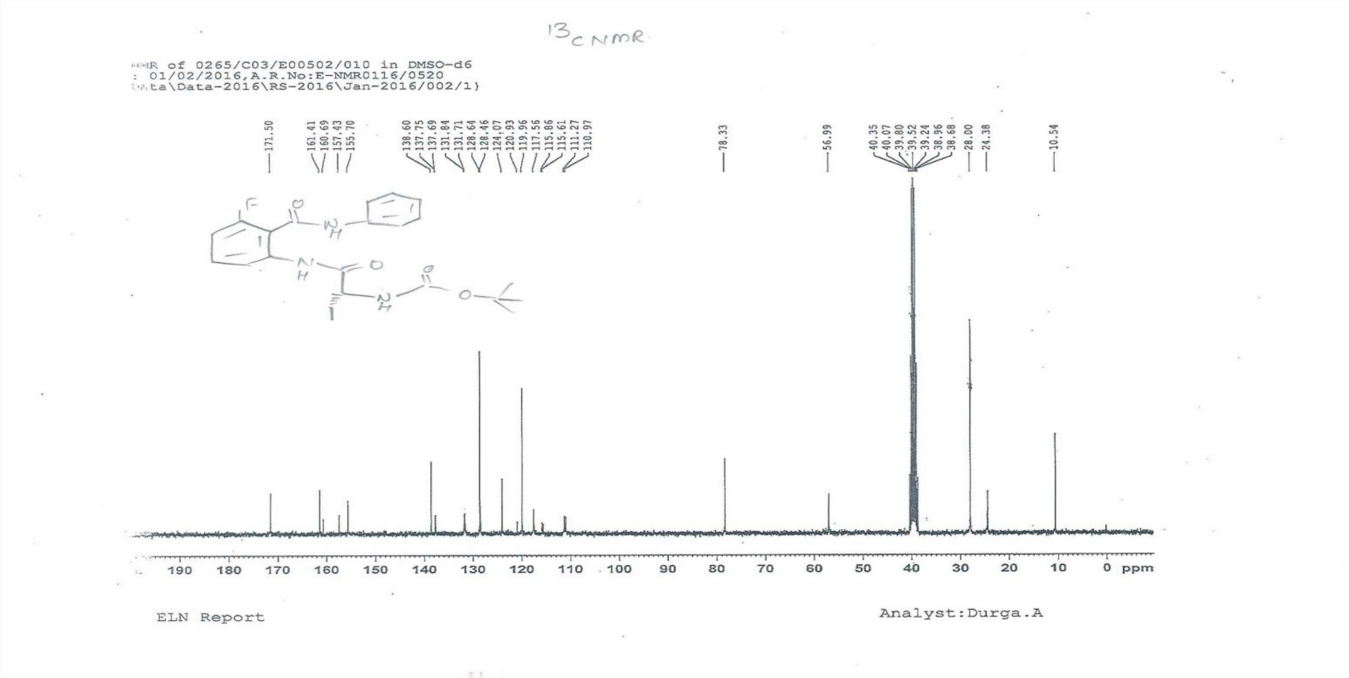

Mass Spectrum of Stage-3

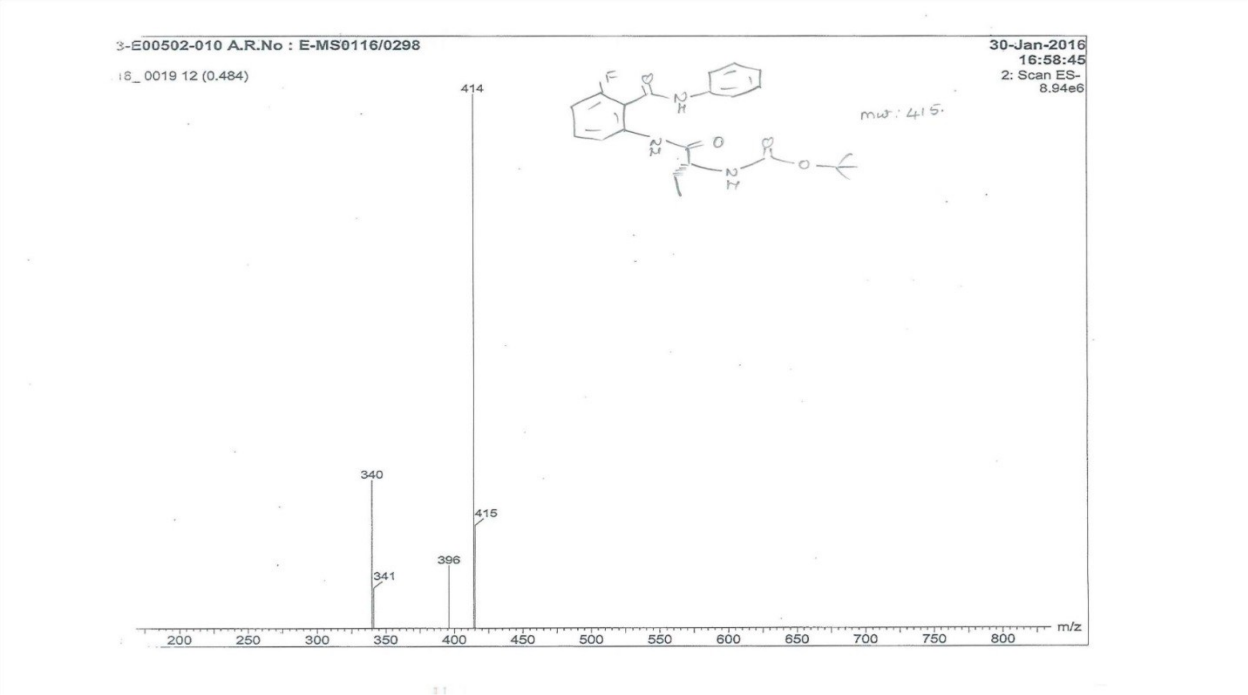

DSC Thermogram of Stage-3

LAURUS Labs

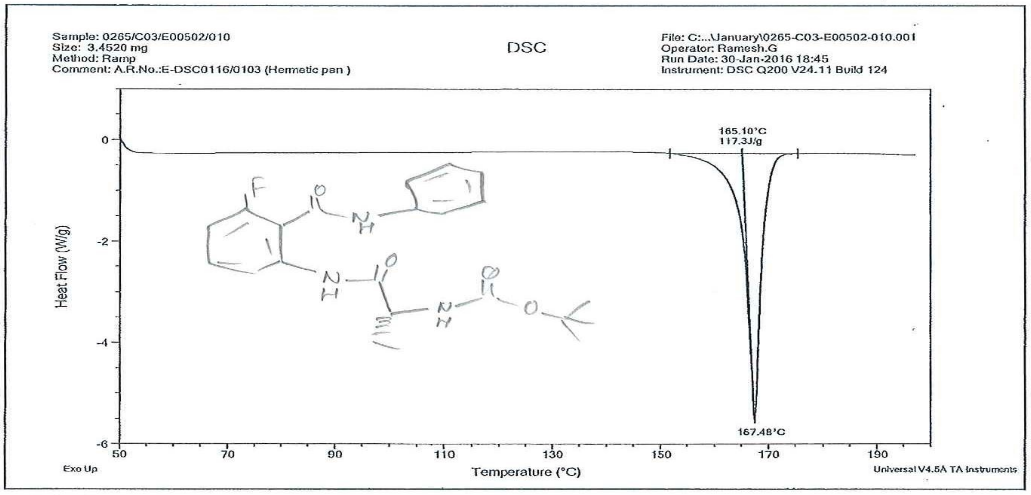

Method Log:

1: Equilibrate at 50.00°C

2: Ramp 10.00°C/min to 200.00°C

3: End of method

Peak Integration

Start	Onset	Maximum	Stop	Area
°C	°C	°C	°C	J/g
151.74	165.10	167.48	175.46	117.3

ELN

HPLC Chromatogram of Stage-3 (Chemical purity)

LAURUS Labs **Analytical Development**

SAMPLE INFORMATION

Sample Name:	0265/C03/E00502/169	Method Id ****	
Sample Type:	Unknown	Acquired By:	Anjaneyuluteam
Vial:	12	Date Acquired:	07/20/2016 11:43:07 IST
Injection #:	1	Date Processed:	07/20/2016 13:52:23 IST
Injection Volume:	10.00 ul	Processing Method	0265_PRoc17
Run Time:	60.0 Minutes	Processing Method Id 5004	
Sample Set Name	0265_C04_60Min_200716	Proc. Chnl. Descr.:	Detector A 245nm
Sample Set ID:	4935	System Name LL_AD_LC_55_176	
Project Name	HYD1_AD-Generics\0265_JULY_	Empower 3 Software Build 3471 SPs Installed:	
Result Id 5014	Result Set Id	Feature Release 2 DB ID: 2487711617	

Auto-Scaled Chromatogram

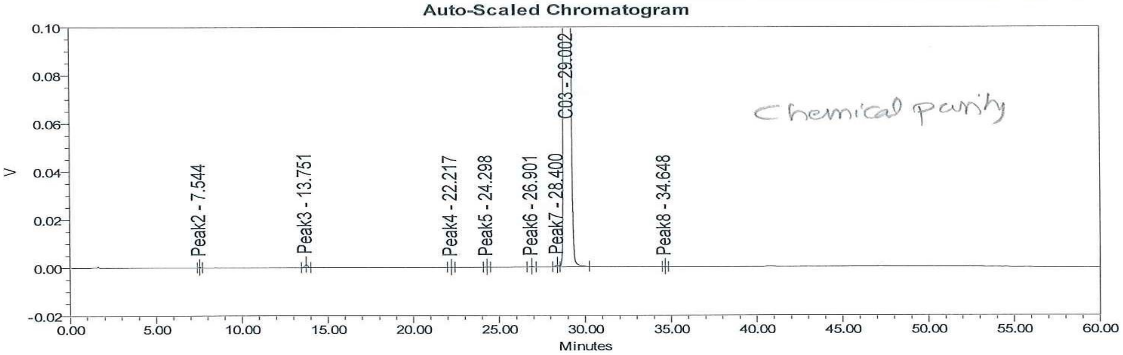

Peak Results

	Name	RT	Area	% Area	RT Ratio
1	Peak2	7.54	1719	0.01	0.26
2	Peak3	13.75	15152	0.09	0.47
3	Peak4	22.22	748	0.00	0.77
4	Peak5	24.30	1885	0.01	0.84
5	Peak6	26.90	3451	0.02	0.93
6	Peak7	28.40	9567	0.06	0.98
7	C03	29.00	1708079[illegible]	99.81	1.00
8	Peak8	34.65	817	0.00	1.19

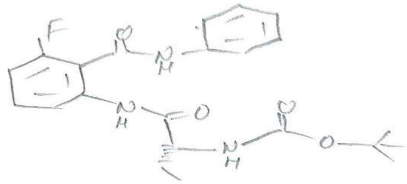

Sample Name: 0265/C03/E00502/169
Date Acquired: 07/20/2016 11:43:07 IST

Signature / Date

HPLC Chromatogram of Stage-3
(Chiral purity)

LAURUS Labs

SAMPLE INFORMATION

Sample Name:	0265/C03/E00502/169	Method Id ****	
Sample Type:	Unknown	Acquired By:	divyakumarteam
Vial:	51	Date Acquired:	07/20/2016 20:14:39 IST
Injection #:	1	Date Processed:	07/21/2016 8:42:26 IST
Injection Volume:	20.00 ul	Processing Method	0265_C03 Rev chiral pro
Run Time:	40.0 Minutes	Processing Method Id 2682	
Sample Set Name	0265_C03 chiral_75_200716	Proc. Chnl. Descr.:	W2489 ChA 245nm
Sample Set ID:	2670	System Name LL_AD_LC_075	
Project Name	HYD1_AD-Generics\0265_July_2	Empower 3 Software Build 3471 SPs Installed:	
Result Id 2687	Result Set Id	Feature Release 2 DB ID: 2487711617	

Auto-Scaled Chromatogram

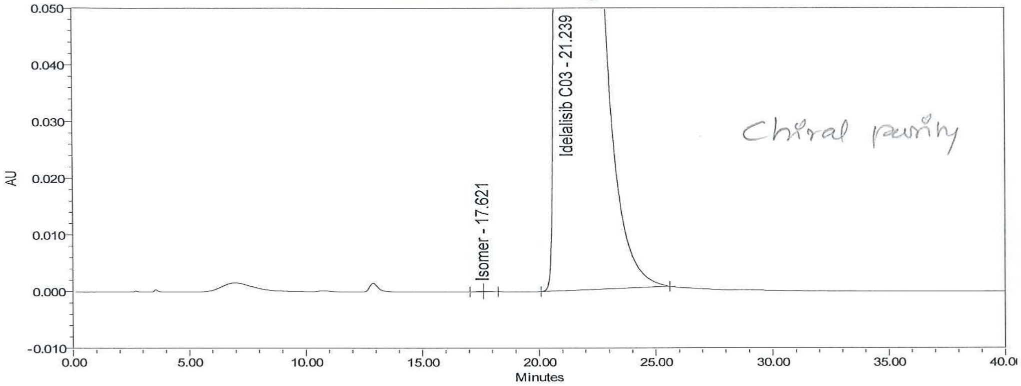

Peak Results

	Name	RT	Area	% Area	RT Ratio	USP Resolution	USP Plate Count	USP Tailing
1	Isomer	17.621	3550	0.009	0.830		4194.4	1.0
2	Idelalisib C03	21.239	3885485[illegible]	99.991		2.41	1973.9	2.0

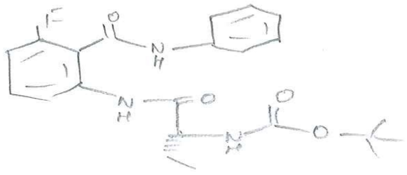

Sample Name: 0265/C03/E00502/169
Date Acquired: 07/20/2016 20:14:39 IST

Signature / Date

Page: 1 of 1

^{1}HNMR Spectrum of stage-4

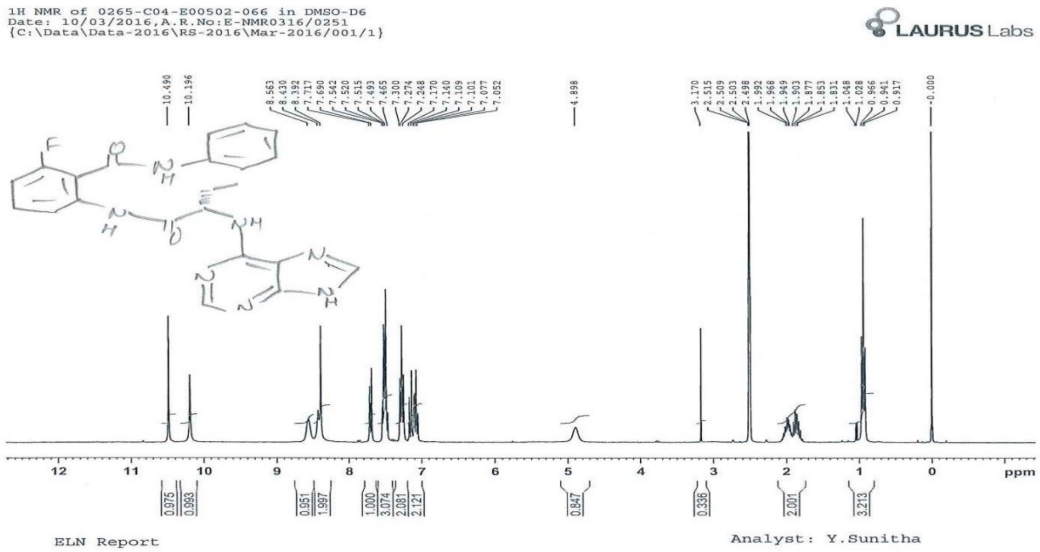

^{13}CNMR Spectrum of stage-4

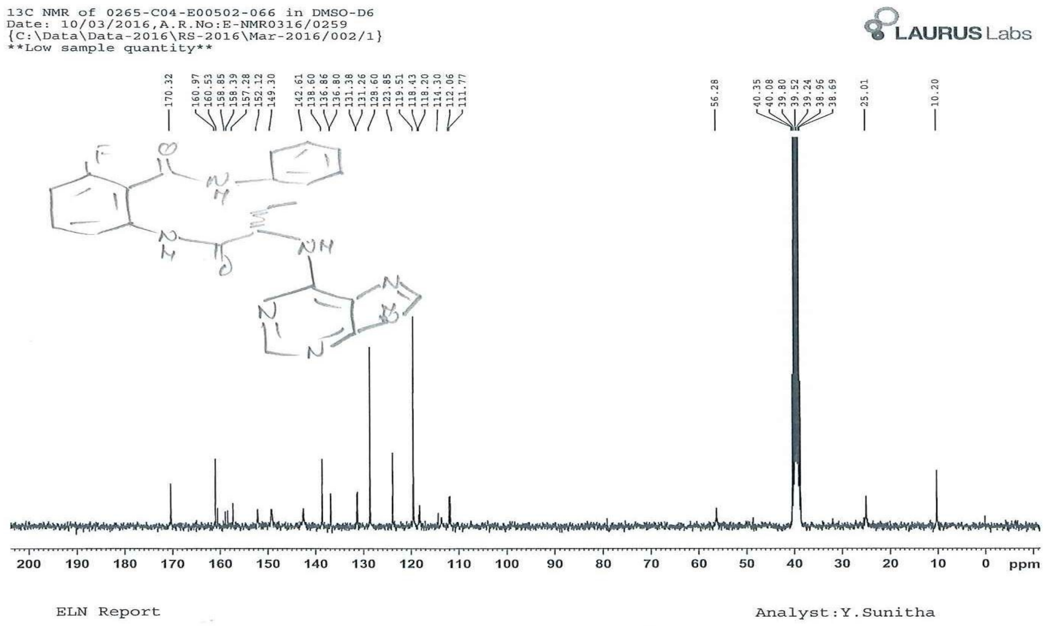

Mass Spectrum of stage-4

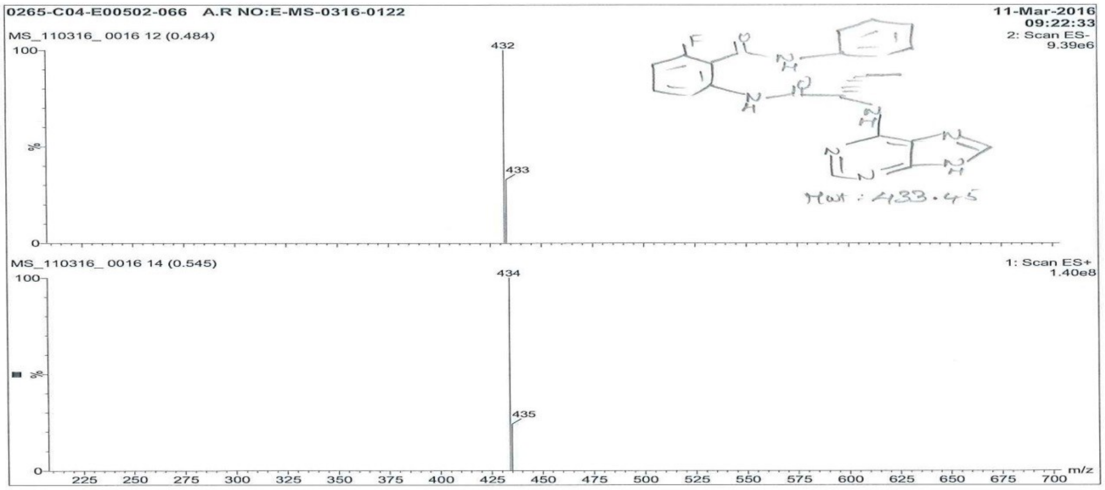

DSC Thermogram of stage-4

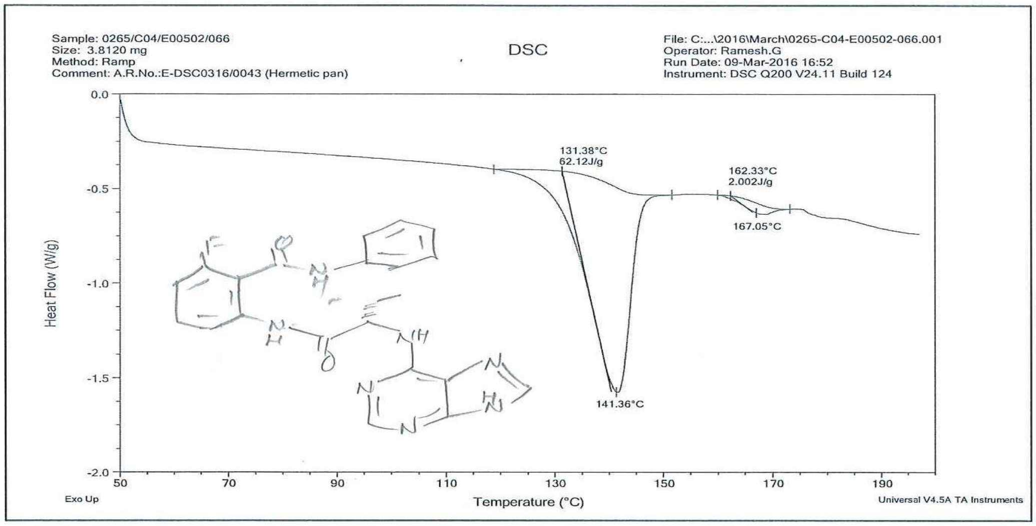

Method Log:

1: Equilibrate at 50.00°C

2: Ramp 10.00°C/min to 200.00°C

3: End of method

Peak Integration

Start	Onset	Maximum	Stop	Area
°C	°C	°C	°C	J/g
118.79	131.38	141.36	151.56	62.12
159.97	162.33	167.05	173.22	2.002

ELN

HPLC Chromatogram of stage-4

LAURUS Labs **Analytical Development**

SAMPLE INFORMATION

Sample Name:	0265/C04/E00502/066	Method Id ****	
Sample Type:	Unknow n	Acquired By:	Anjaneyuluteam
Vial:	7	Date Acquired:	3/10/2016 3:12:23 AM IST
Injection #:	1	Date Processed:	3/10/2016 8:57:13 AM IST
Injection Volume:	10.00 ul	Processing Method:	0265_PRO1
Run Time:	80.0 Minutes	Processing Method Id 1327	
Sample Set Name:	100316_C05	Proc. Chnl. Descr.:	Detector A 268nm
Sample Set ID:	1234	System Name AL_AD_LC_32_125	
Project Name	HYD1_AD-Generics\0265_MAR_[illegible]	Empow er 3 Softw are Build 3471 SPs Installed: Feature	
Result Id 1337	Result Set Id	Release 2 DB ID: 2487711617	

Auto-Scaled Chromatogram

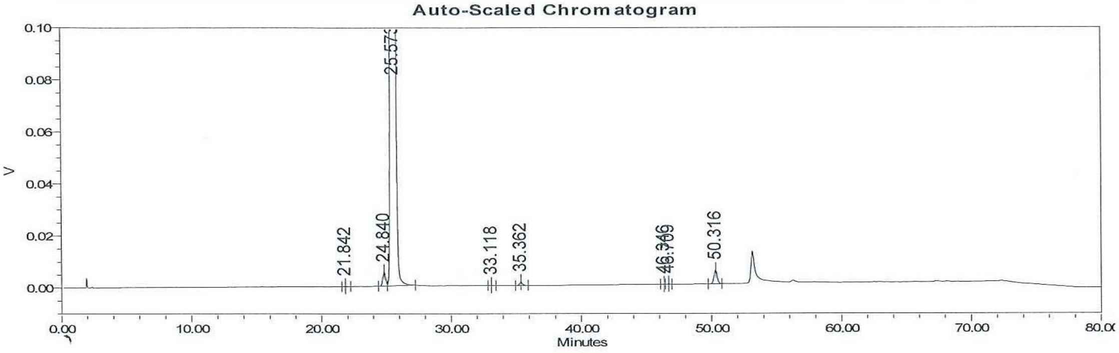

Peak Results

	Name	RT	Area	% Area	RT Ratio
1	Peak2	21.842	2361	0.010	0.854
2	Peak3	24.840	81196	0.360	0.971
3	C04	25.573	22378645	99.117	1.000
4	Peak4	33.118	1144	0.005	1.295
5	Peak5	35.362	20213	0.090	1.383
6	Peak6	46.346	515	0.002	1.812
7	Peak7	46.709	1828	0.008	1.827
8	Peak8	50.316	92194	0.408	1.968

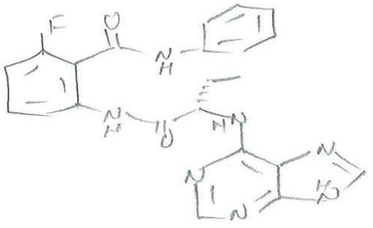

Sample Name: 0265/C04/E00502/066
Date Acquired: 3/10/2016 3:12:23 AM IST

Signature / Date

^{1}HNMR Spectrum of stage-5/ Idelalisib

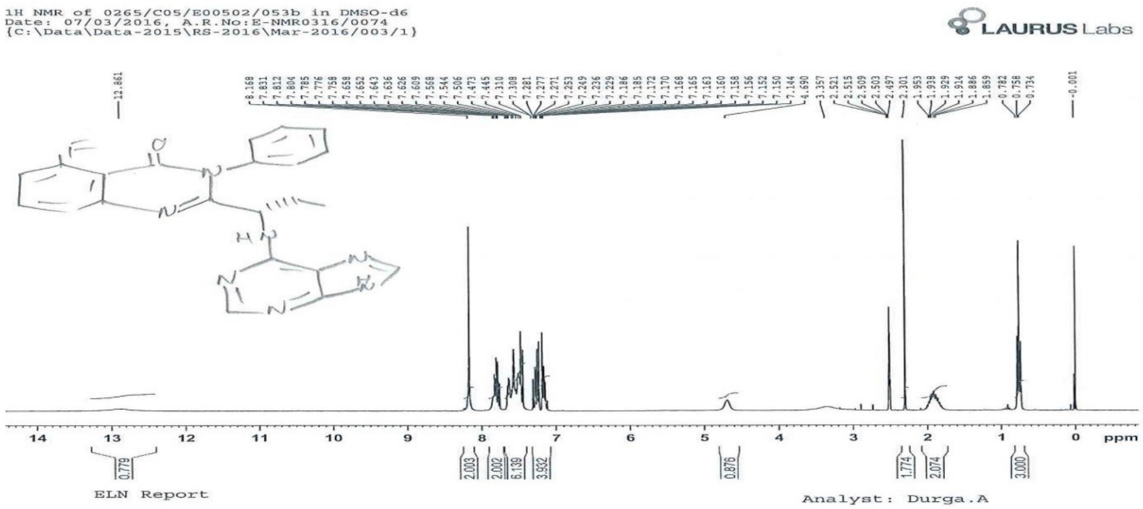

^{13}CNMR Spectrum of stage-5/ Idelalisib

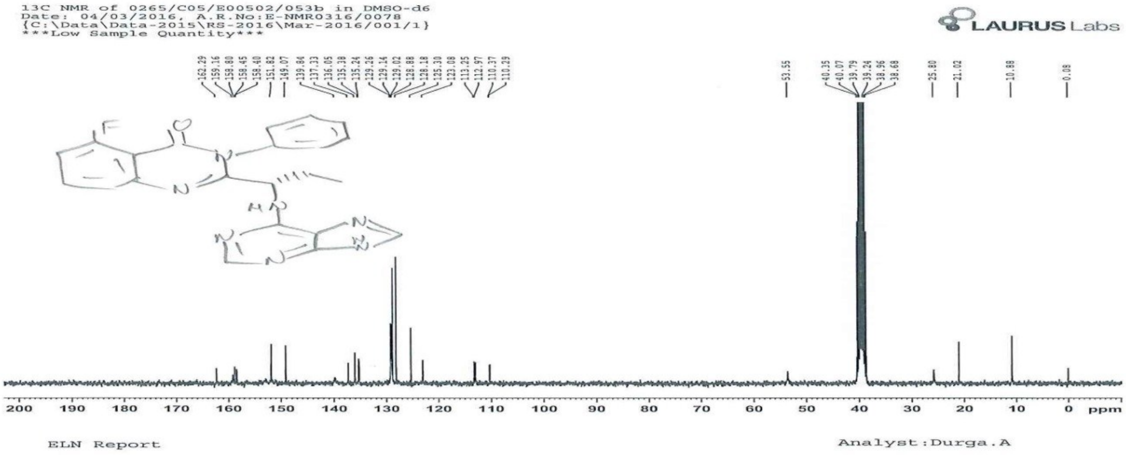

Mass Spectrum of stage-5/ Idelalisib

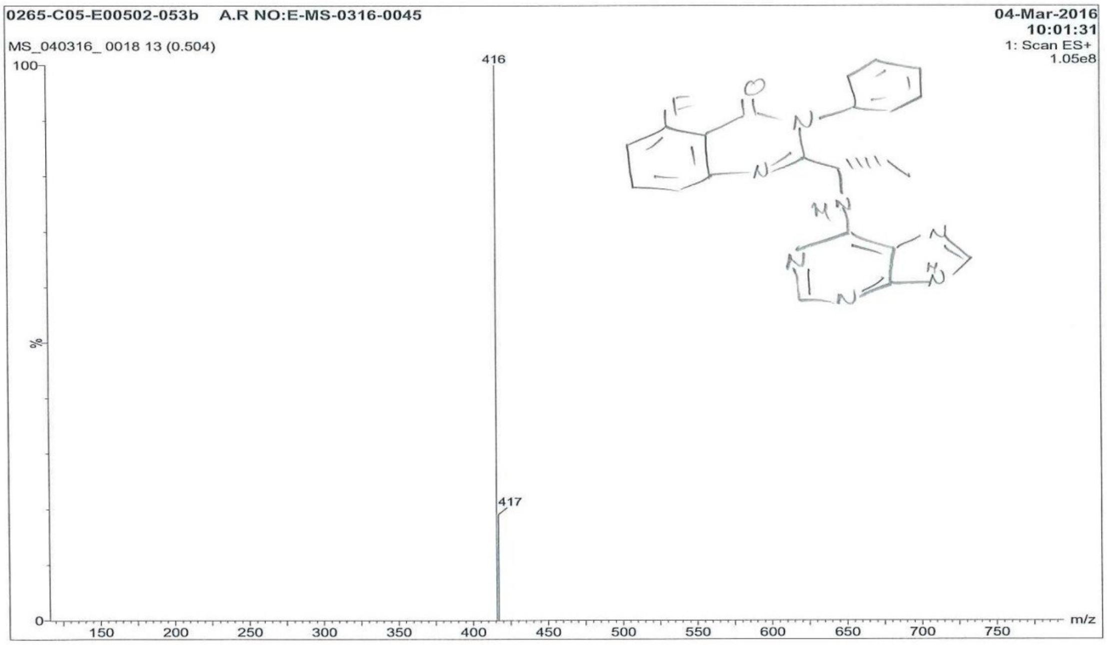

DSC Thermogram of stage-5/ Idelalisib

LAURUS Labs

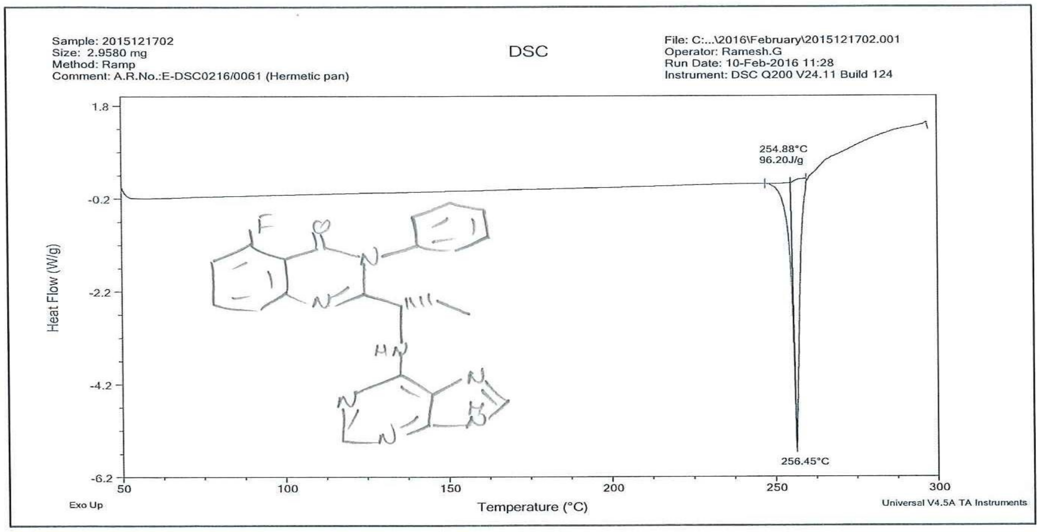

Method Log:

1: Equilibrate at 50.00°C

2: Ramp 10.00°C/min to 300.00°C

3: End of method

Peak Integration

Start	Onset	Maximum	Stop	Area
°C	°C	°C	°C	J/g
247.21	254.88	256.45	259.85	96.20

ELN

HPLC Chromatogram of stage-5/ Idelalisib (Chemical purity)

LAURUS Labs **Analytical Development**

SAMPLE INFORMATION

Sample Name:	0265/C05/E00502/149
Sample Type:	Unknown
Vial:	2:3
Injection #:	1
Injection Volume:	10.00 ul
Run Time:	80.0 Minutes
Sample Set Name	0265_C05_80Min_020716
Sample Set ID:	1238
Project Name	HYD1_AD-Generics\0265_JULY_
Result Id 1274	Result Set Id

Method Id ****	
Acquired By:	Anjaneyuluteam
Date Acquired:	07/02/2016 12:36:03 IST
Date Processed:	07/02/2016 14:15:50 IST
Processing Method	0265_PRo1
Processing Method Id 1262	
Proc. Chnl. Descr.:	Detector A 268nm
System Name LL_AD_LC_56_177	
Empower 3 Software Build 3471 SPs Installed:	
Feature Release 2 DB ID: 2487711617	

Auto-Scaled Chromatogram

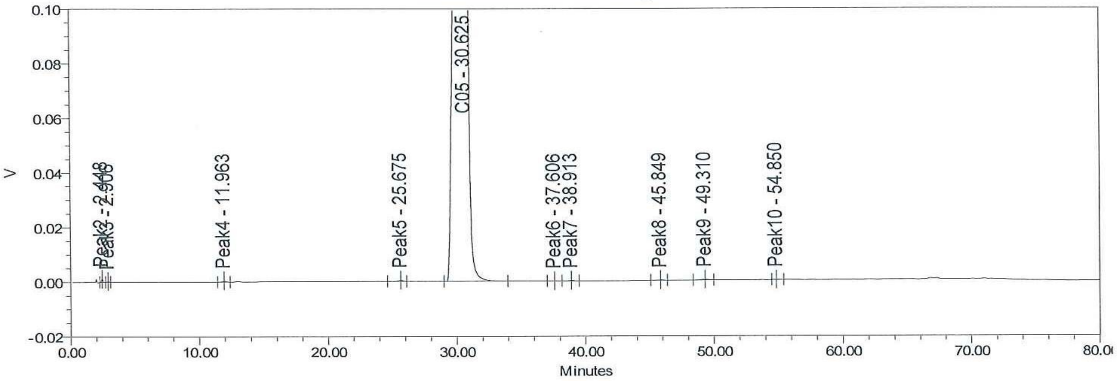

Peak Results

Name	RT	Area	% Area	RT Ratio
Peak2	2.45	8615	0.02	0.08
Peak3	2.91	2627	0.01	0.09
Peak4	11.96	7442	0.02	0.39
Peak5	25.68	13446	0.03	0.84
C05	30.63	4540977[illegible]	99.87	1.00
Peak6	37.61	2321	0.01	1.23
Peak7	38.91	5640	0.01	1.27
Peak8	45.85	4815	0.01	1.50
Peak9	49.31	9733	0.02	1.61
Peak10	54.85	2499	0.01	1.79

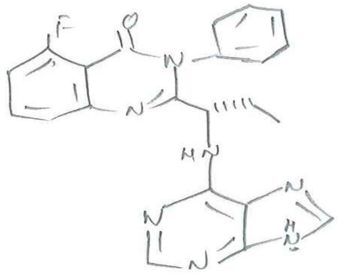

Sample Name: 0265/C05/E00502/149
Date Acquired: 07/02/2016 12:36:03 IST

Signature / Date

HPLC Chromatogram of stage-5/ Idelalisib

(Chiral purity)

LAURUS Labs **Analytical Development**

SAMPLE INFORMATION

Sample Name:	0265/C05/E00502/149	Method Id ****	
Sample Type:	Unknow n	Acquired By:	divyakumarteam
Vial:	1	Date Acquired:	7/2/2016 11:00:00 AM IST
Injection #:	1	Date Processed:	7/2/2016 2:30:27 PM IST
Injection Volume:	10.00 ul	Processing Method:	0265_C05_chiral pro
Run Time:	40.0 Minutes	Processing Method Id	1024
Sample Set Name:	0265_C05_chiral	Proc. Chnl. Descr.:	W2489 ChA 268nm
Sample Set ID:	1007	System Name	LL_AD_LC_075
Project Name	HYD1_AD-Generics\0265_July_2	Empow er 3 Softw are Build 3471 SPs Installed: Feature	
Result Id 1031	Result Set Id	Release 2 DB ID: 2487711617	

Auto-Scaled Chrom atogram

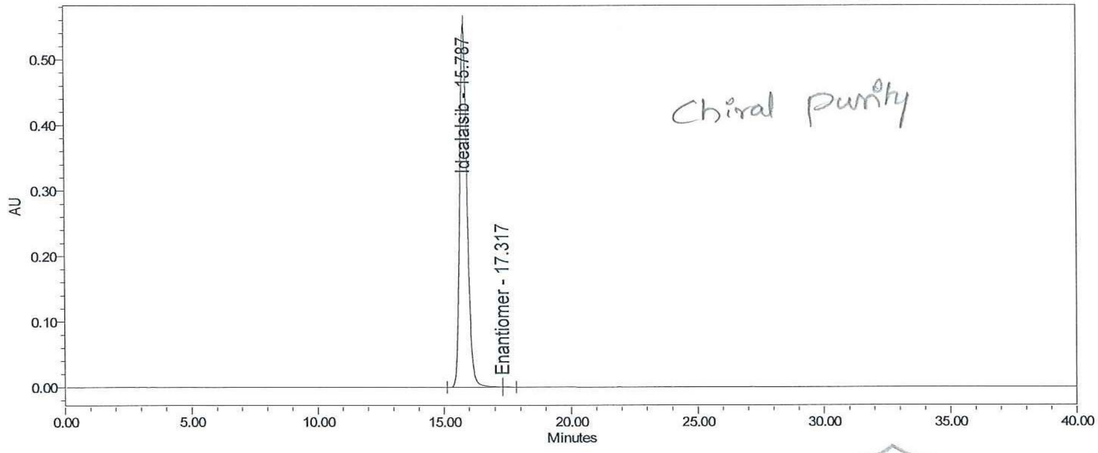

Peak Results

	Name	RT	Area	% Area	RT Ratio
1	Idealalsib	15.787	11482128	99.951	
2	Enantiomer	17.317	5666	0.049	

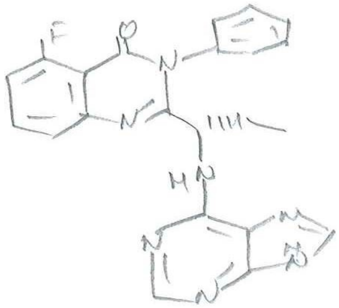

Signature / Date
